# KIF5A downregulation in spinal muscular atrophy links axonal regeneration defects with ALS

**DOI:** 10.1172/jci.insight.197941

**Published:** 2026-03-26

**Authors:** Tetsuya Akiyama, Yi Zeng, Caiwei Guo, Olivia Gautier, Lauren Koepke, Heankel Lyons, Elana Molotsky, Juliane S. Bombosch, Odilia Sianto, Jay P. Ross, Phuong Hoang, Luke Zhao, Cole Spencer, Charlotte J. Sumner, Michelle Monje, John W. Day, Aaron D. Gitler

**Affiliations:** 1 Department of Genetics, Stanford University School of Medicine, Stanford, California, USA.; 2 Stanford Neurosciences Graduate Program,; 3 Department of Neurology and Neurological Sciences, and; 4 Stem Cell Biology and Regenerative Medicine, Stanford University, Stanford, California, USA.; 5 Department of Neurology and; 6 Department of Neuroscience, Johns Hopkins University School of Medicine, Baltimore, Maryland, USA.; 7 Howard Hughes Medical Institute and; 8 Department of Neurology and Clinical Neurosciences, Stanford University, Stanford, California, USA.; 9 Biohub – San Francisco, San Francisco, California, USA.; 10 The Phil and Penny Knight Initiative for Brain Resilience, Stanford University, Stanford, California, USA.

**Keywords:** Genetics, Neuroscience, Neuromuscular disease

## Abstract

Spinal muscular atrophy (SMA) is a devastating neuromuscular disorder caused by mutations in the survival motor neuron 1 (*SMN1*) gene leading to decreased SMN protein levels and motor neuron dysfunction. SMN-restoring therapies offer clinical benefit, but the downstream molecular consequences of SMN reduction remain incompletely understood. SMN deficiency resulted in downregulation of kinesin heavy chain isoform 5A (KIF5A) in human neurons and in a mouse model of SMA. SMN associated with *KIF5A* mRNA and contributed to its stability. Reduced SMN levels impaired axon regeneration, which was rescued by KIF5A overexpression. Because KIF5A has also been connected to ALS, these findings provide evidence of a molecular link between SMA and ALS pathophysiology, highlighting KIF5A as an SMN-regulated factor. Our findings suggest that SMN-independent interventions targeting KIF5A could represent a complementary therapeutic approach for SMA and other motor neuron diseases.

## Abstract 2

SMN deficiency impairs axon regeneration by destabilizing *KIF5A* mRNA, linking SMA to ALS and suggesting KIF5A as a potential therapeutic target

## Graphical abstract

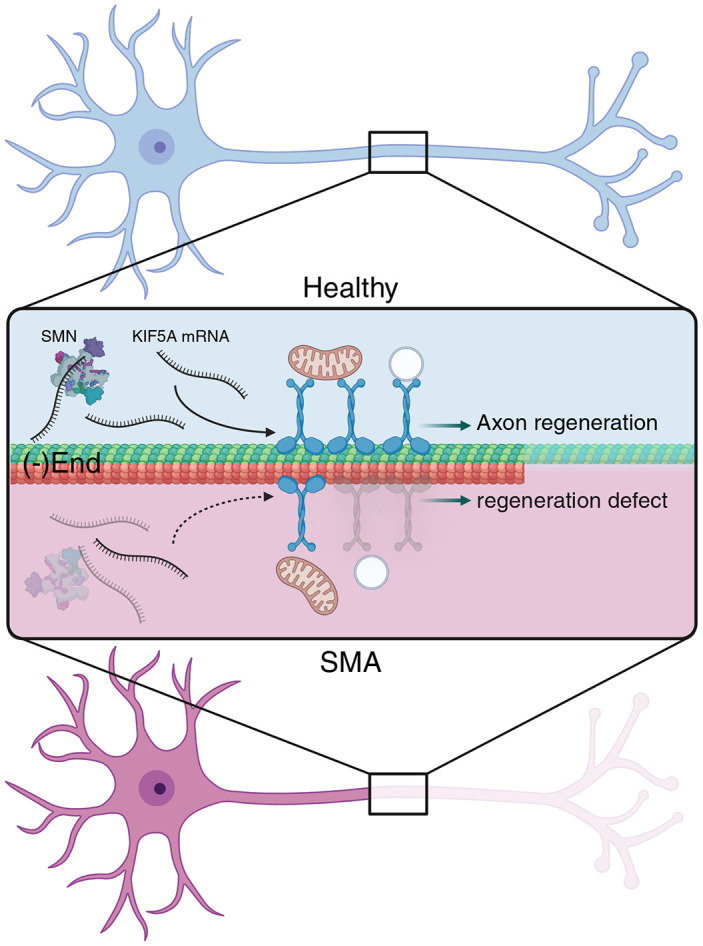

## Introduction

Spinal muscular atrophy (SMA) is the most common inherited cause of infant mortality (1–3). SMA is caused by homozygous loss-of-function mutations or deletions in the *SMN1* gene, which encodes survival motor neuron (SMN) protein. Loss of SMN causes lower motor neuron degeneration, which leads to progressive muscle weakness, paralysis, and eventually death, most often within the first 2 years of life without treatment (4, 5). SMN is a highly conserved ubiquitous protein that plays critical roles in multiple cellular processes essential for motor neuron survival and function, including the assembly of small nuclear ribonucleoproteins (snRNPs), pre-mRNA splicing, RNA metabolism, and axonal mRNA transport (6–9).

Humans have a paralogous gene, *SMN2*, which although nearly identical to *SMN1* has a single-nucleotide substitution that affects pre-mRNA splicing resulting in frequent exclusion of exon 7 with production of unstable SMN protein (4). Typically, patients with SMA have zero functioning copies of *SMN1*, and the severity of the disease inversely correlates with the copy number of the *SMN2* gene. Humans carry variable numbers of *SMN2* copies (10), and the more copies of *SMN2*, the milder the clinical features. This observation has led to 3 different therapeutic strategies, which have significantly improved clinical outcomes by increasing SMN protein levels (11–13). The first approach uses an antisense oligonucleotide (ASO) that blocks SMN2 intron 7 splicing repressors, thus improving exon 7 inclusion in *SMN2* transcripts and increasing SMN protein levels (4, 5, 14, 15). Another approach delivers adeno-associated viral vectors intravenously to transduce CNS neurons and deliver an episomal *SMN* genetic construct (12) that expresses high levels of SMN protein. Finally, an orally administered small-molecule *SMN2* splicing modifier such as risdiplam promotes *SMN2* exon 7 inclusion by binding the *SMN2* pre-mRNA at 2 sites and stabilizing productive spliceosome assembly, thereby increasing full-length SMN protein production (16, 17).

All 3 of these approaches are now approved by the US FDA and are being used in clinical practice. Simply put, they have changed the natural history for this once-fatal disorder (3). But these therapies do not completely eliminate disease-related deficits or prevent their progression, particularly when treatment initiation is delayed or in patients with severe SMA phenotypes (18–20). Moreover, with the splice modulating approaches, there is a natural ceiling effect based on *SMN2* pre-mRNA expression levels (21). For gene therapy, there is the potential for long-term toxicity from overexpressing SMN (22), raising questions about its long-term safety and efficacy. Together, these limitations underscore the need to identify complementary therapeutic targets that act independently of SMN restoration. To this end, here we focused on defining the downstream cellular and molecular consequences of SMN reduction.

Kinesin family member 5A (KIF5A) is a neuron-specific motor protein essential for axonal transport of various cargos, including mitochondria, synaptic vesicles, and mRNAs (23). Mutations in *KIF5A* have been identified as causative factors in adult-onset motor neuron diseases such as amyotrophic lateral sclerosis (ALS) (24, 25), axonal neuropathy (Charcot-Marie-Tooth disease type 2; CMT2) (26), hereditary spastic paraplegia (HSP) (27, 28), and adult-onset distal SMA (29), highlighting its critical role in maintaining motor neuron integrity. KIF5A-null human motor neurons exhibit impaired axonal regeneration, further underscoring the critical role of KIF5A in axonal repair (30). In addition to motor neuron diseases, KIF5A levels have been reported to be downregulated in models of Alzheimer’s disease (31), Parkinson’s disease (32), and multiple sclerosis (33). Furthermore, KIF5A downregulation has been observed in *SOD1* mutant astrocytes, suggesting its broader role in neurodegeneration and glial dysfunction (34). These findings suggest that impaired axonal regeneration can represent a shared feature observed across multiple neurological disorders. Despite the established role of KIF5A in motor neuron health, its potential involvement in SMA pathogenesis has not been previously explored.

In this manuscript, while we were analyzing RNA-seq data to investigate the downstream molecular consequences of SMN deficiency, we discovered a dramatic reduction in *KIF5A* expression in SMN-deficient human motor neurons. We confirmed reduced KIF5A expression in spinal motor neurons in 2 SMA mouse models and in SMA patient–derived motor neurons, indicating a conserved pathomechanism. Importantly, we show that restoring KIF5A expression is sufficient to rescue axonal regeneration defects caused by SMN deficiency. We provide evidence that SMN associates with *KIF5A* mRNA and contributes to its stability, suggesting a potentially novel mechanism by which SMN deficiency leads to impaired axonal regeneration. Together, this work reveals KIF5A as a therapeutic target that could complement existing SMN-restoring therapies. Our findings also reveal a molecular link between SMA and ALS, providing insights into the mechanisms underlying motor neuron degeneration.

## Results

**Figure 1:**
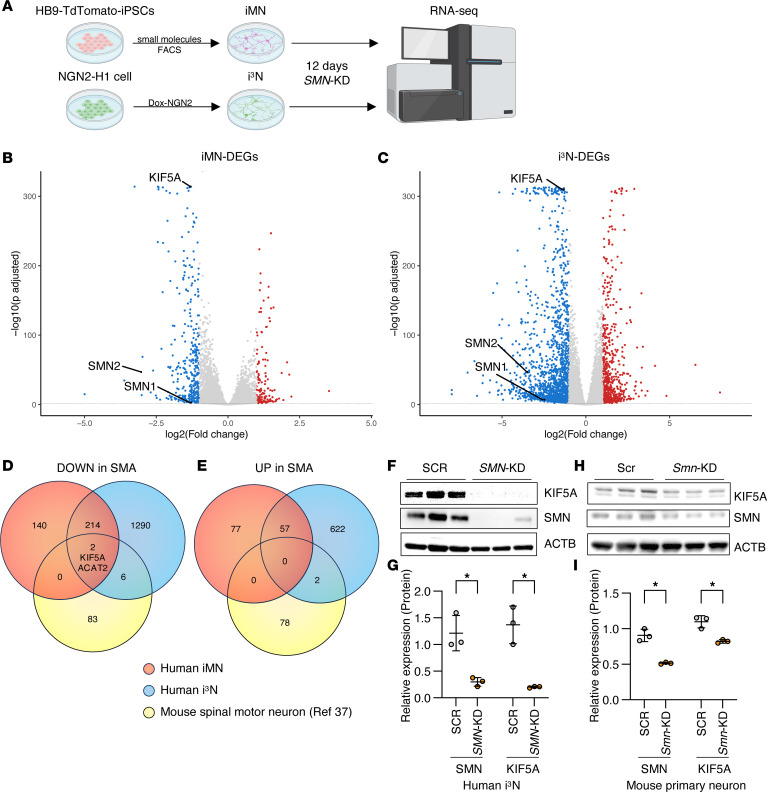
SMN deficiency downregulates KIF5A expression. (**A**) Two types of human neurons were generated and analyzed. Td-Tomato^+^ motor neurons (iMNs) were derived from human iPSCs by small molecule treatment and purified using fluorescence-activated cell sorting (FACS) under the control of the HB9 promoter. In parallel, i^3^Neurons (i^3^Ns) were produced by doxycycline-induced NGN2 expression from isogenic, integrated human H1 ESCs (Supplemental Table 1). Both iMNs and i^3^Ns were subsequently transduced with lentivirus carrying shRNA targeting *SMN1/2* (*SMN*-KD), followed by RNA-seq. (**B** and **C**) Volcano plots showing differentially expressed genes (DEGs) in iMNs (**B**) and i^3^Ns (**C**) following *SMN*-KD. Each plot displays log_2_ fold change (*x* axis) versus negative log10 adjusted *P*-value (*y* axis). Genes significantly upregulated (red dots) or downregulated (blue dots) are defined by adjusted *P* < 0.01 and |log_2_ fold change| > 1. (**D** and **E**) Venn diagrams integrating RNA-seq datasets from iMNs (red), i^3^Ns (blue), and laser-microdissected motor neurons from SMA model mice (yellow) (37). Downregulated DEGs (**D**) and upregulated DEGs (**E**) were defined by adjusted *P* < 0.01 and |log_2_ fold change| > 1. Gene lists are provided in Supplemental Table 2. *KIF5A* was identified as a commonly downregulated gene. (**F**–**I**) Western blot analysis of i^3^N lysates following *SMN*-KD (**F**) and of primary mouse cortical neurons treated with siRNAs targeting *Smn* (**H**), confirming reduced SMN and KIF5A protein levels. Quantification of SMN and KIF5A protein levels relative to scrambled controls (SCR or Scr) is shown in **G** and **I**. Data represent 3 independent experiments. Statistical analysis was performed using an unpaired 2-tailed *t* test with Welch’s correction.

To investigate the molecular mechanisms underlying SMA, we performed RNA-seq of human neurons following *SMN* knockdown (*SMN*-KD). We used human neurons derived from either induced pluripotent stem cells (iPSCs) or human embryonic stem (ES) cells (Supplemental Table 1; supplemental material available online with this article; https://doi.org/10.1172/jci.insight.197941DS1) and 2 distinct systems to induce neuronal differentiation. To make motor neurons, we used HB9–Td-Tomato iPSCs (35), in which Td-Tomato was expressed under the HB9 promoter, allowing for the isolation of motor neurons (iMNs) via FACS (Figure 1A). To generate excitatory glutamatergic neurons, we used H1 ES cells engineered for inducible NGN2 expression (i^3^Neurons; i^3^Ns) (36). We targeted both cell types for *SMN*-KD using lentiviral shRNA against *SMN1/2*. Following 12 days of *SMN*-KD, we harvested RNA for RNA-seq (Figure 1A).

As expected, *SMN1* and *SMN2* RNA levels were significantly reduced (Figure 1, B and C). In iMNs, *SMN*-KD resulted in downregulation of 362 genes and upregulation of 138 genes (Figure 1, D and E, and Supplemental Table 2). In i^3^Ns, 1,530 genes were downregulated, and 688 genes were upregulated (Figure 1, D and E, Supplemental Table 2). Notably, 216 genes were commonly downregulated, and 57 genes were commonly upregulated in both cell types (Figure 1, D and E, and Supplemental Table 2). To narrow the list of SMN targets, we compared our set of up- and downregulated genes to a previously reported RNA-seq dataset of motor neurons isolated by laser microdissection from the spinal cord of an SMA mouse model (Figure 1, D and E, and Supplemental Table 2) (37). There were only 2 genes, *ACAT2* and *KIF5A*, downregulated in all 3 datasets (Figure 1D). qPCR and immunoblot analysis confirmed that SMN reduction resulted in decreased KIF5A mRNA and protein levels in human i^3^Ns (Figure 1, F and G, and Supplemental Figure 1, A–C) and iMNs (Supplemental Figure 1, D–F), whereas ACAT2 protein levels remained unchanged despite reduced mRNA expression (Supplemental Figure 1, A–F). Although *ACAT2* mRNA levels were reduced in SMA neurons, ACAT2 protein levels remain unchanged under the conditions tested, suggesting buffering at the posttranscriptional or protein-stability level. KIF5A is a neuron-enriched member of the kinesin-1 family together with KIF5B and KIF5C (38). Among these family members, KIF5A showed the most robust and consistent reduction following SMN depletion (Supplemental Figure 1, A–F). We further confirmed *KIF5A* mRNA reduction using single-molecule FISH (smFISH) by directly counting mRNA granules in *SMN*-KD i^3^Ns (Supplemental Figure 1, G–I). To test if KIF5A downregulation also occurs in mouse neurons and to assess the conservation of this effect across species, we performed siRNA-mediated *Smn*-KD in embryonic mouse primary cortical neurons. This resulted in a robust and reproducible reduction in both SMN and KIF5A protein levels (Figure 1, H and I). These findings validate the transcriptomic data and indicate that the downregulation of KIF5A is a conserved and specific response to SMN deficiency.

**Figure 2:**
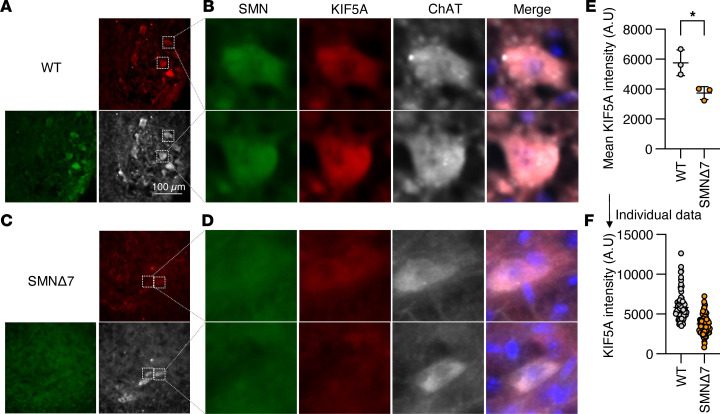
KIF5A is downregulated in SMA model mice. (**A**–**D**) IHC analysis of whole spinal cord sections from P10 SMNΔ7 mice (**A** and **B**) and age-matched control mice (**C** and **D**). SMN is shown in green, KIF5A in red, and the motor neuron marker ChAT in gray. Two motor neurons within the white boxed regions in **A** and **C** are shown at higher magnification in **B** and **D**, respectively. (**E** and **F**) Spinal motor neurons from SMA model mice exhibited reduced KIF5A immunoreactivity. (**E**) Quantification of KIF5A intensity was performed by averaging measurements from more than 20 motor neurons sampled across nonconsecutive sections from 3 biologically independent mice per group. Statistical comparison was performed using an unpaired *t* test (2 tailed) on per-animal averages. (**F**) Individual KIF5A intensity values for all quantified motor neurons are shown. Scale bar: 100 μm (**A** and **C**).

To validate our in vitro results in vivo, we first examined KIF5A expression in the spinal cord of an SMA mouse model known as SMNΔ7. These mice carry a homozygous deletion of the endogenous mouse *Smn* gene and are transgenic for 2 copies of the human *SMN2* gene and approximately 12 copies of a human S*MN* cDNA lacking exon 7 (referred to as SMNΔ7). This combination results in a severe SMA phenotype that closely resembles the human disease (39, 40). Compared with 10-day-old SMNΔ7 age-matched control animals, spinal motor neurons from SMA mice exhibited significantly reduced KIF5A staining (Figure 2, A–F, and Supplemental Figure 2, A–D). Notably, a subset of motor neurons in SMA mice retained detectable KIF5A expression (Supplemental Figure 2, C and D), resulting in partial overlap of KIF5A intensity values between SMA and control groups (Figure 2F). Consistent with this observation, residual SMN expression was also preserved in some motor neurons (Supplemental Figure 2, C and D). Importantly, motor neurons with higher SMN intensity tended to maintain higher levels of KIF5A, revealing a positive correlation between SMN and KIF5A expression that was not observed with ChAT staining (Supplemental Figure 2E).

Because SMNΔ7 mice exhibit a rapidly progressing, end-stage phenotype by P10, we further examined *Smn^2B/–^* mice, a milder SMA model with delayed disease onset and longer survival (41, 42). Using a genetic strategy to fluorescently label motor neuron nuclei, we analyzed KIF5A expression in spinal motor neurons in *Smn^2B/–^* mice at P17. P17 represents a symptomatic stage in this model, during which motor deficits are emerging but mice have not yet reached end stage, allowing assessment of molecular alterations prior to advanced disease stage (41, 42). Our analysis revealed a directionally consistent but modest trend toward reduced KIF5A expression, supporting the overall direction of the changes observed in the severe SMA model (Supplemental Figure 2, F–J).

**Figure 3:**
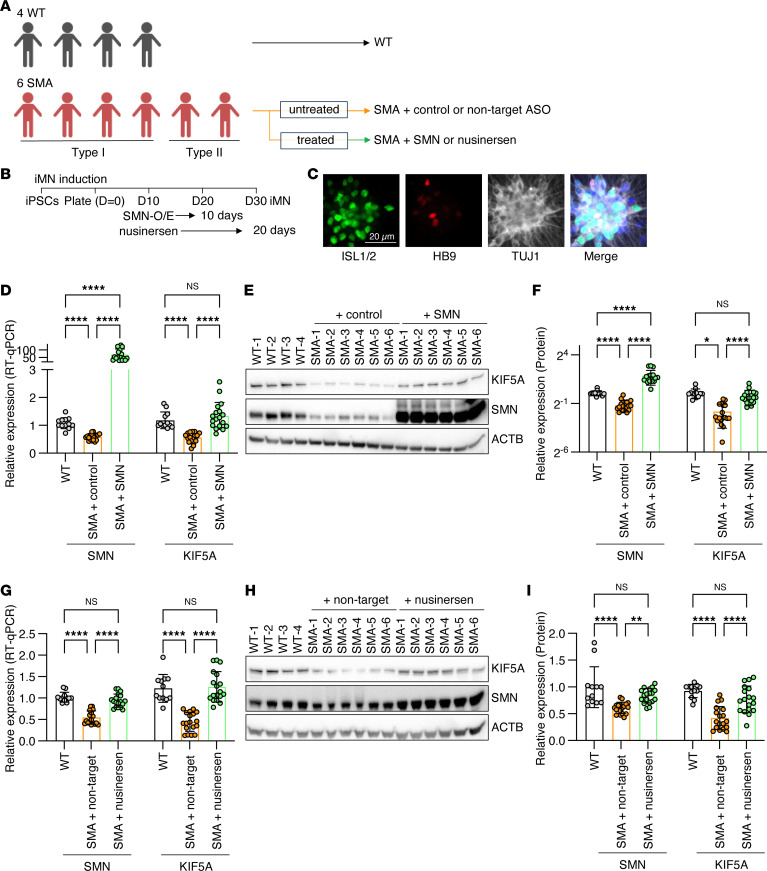
KIF5A is downregulated in SMA patient–derived iPSC motor neurons. (**A**–**C**) Experimental design and characterization of SMA patient–derived motor neurons. (**A**) Motor neurons were differentiated from 4 healthy donor and 6 SMA patient iPSC lines using small-molecule protocols (cell line information in Supplemental Table 1). (**B**) Schematic of the differentiation and treatment timeline. SMN overexpression was induced by lentiviral transduction on day 10 after plating, with samples collected on day 20. For nusinersen treatment, administration started on day 10, and samples were collected on day 30. (**C**) Representative immunocytochemistry images at day 20 showing βIII-tubulin, HB9, and ISL1/2 staining. Additional images and quantifications are shown in Supplemental Figure 3. Scale bar: 20 μm. (**D**–**F**) Restoration of KIF5A expression by SMN overexpression in SMA patient–derived motor neurons. (**D**) qPCR analysis of SMN and *KIF5A* mRNA levels in WT and SMA motor neurons transduced with empty vector (+ control) or SMN-expressing lentivirus (+ SMN). (**E**) Representative Western blots of SMN and KIF5A in WT and SMA-iMNs. (**F**) Quantification of SMN and KIF5A protein levels normalized to ACTB and expressed relative to WT. Individual data points are shown in Supplemental Figure 4D. (**G**–**I**) Effects of nusinersen on SMN and KIF5A expression in SMA patient–derived motor neurons. (**G**) qPCR analysis of *SMN* and *KIF5A* mRNA levels following nusinersen or control ASO treatment, with WT samples included for comparison. (**H**) Representative Western blots of SMN and KIF5A following nusinersen treatment. (**I**) Quantification of SMN and KIF5A protein levels normalized to ACTB. Individual data points are shown in Supplemental Figure 4H. All experiments were performed in triplicate with 3 biologically independent experiments.

Next, to validate our findings from *SMN*-KD models, we examined KIF5A expression in motor neurons derived from SMA patient iPSCs. We utilized iPSC lines from 4 healthy donors and 6 lines from patients with SMA (Figure 3A and Supplemental Table 1, which includes patient demographic details, including *SMN2* copy number). We differentiated iPSCs from 4 healthy donors and 6 patients with SMA into motor neurons with comparable differentiation efficiencies (Figure 3, B and C, and Supplemental Figure 3, A–C). Compared with the 4 healthy donor lines, all 6 SMA patient–derived motor neurons exhibited reduced levels of both SMN and KIF5A mRNA and proteins (Figure 3, D–F). Importantly, upregulation of SMN using a lentivirus was sufficient to restore KIF5A protein levels (Figure 3, D–F), providing evidence that SMN directly regulates KIF5A levels. We also tested an alternative SMN-restoration strategy using the exon 7 splice-skipping ASO, nusinersen, on the SMA patient–derived motor neurons. After 20 days of treatment, we observed consistent increase in SMN expression and restoration of KIF5A levels (Figure 3, G–I), whereas effects on other kinesin-1 family members were limited or context dependent (Supplemental Figure 4, A–H). Together, these data support KIF5A reduction as an SMN-linked molecular change in SMA, although contributions from disease progression in vivo cannot be excluded.

**Figure 4:**
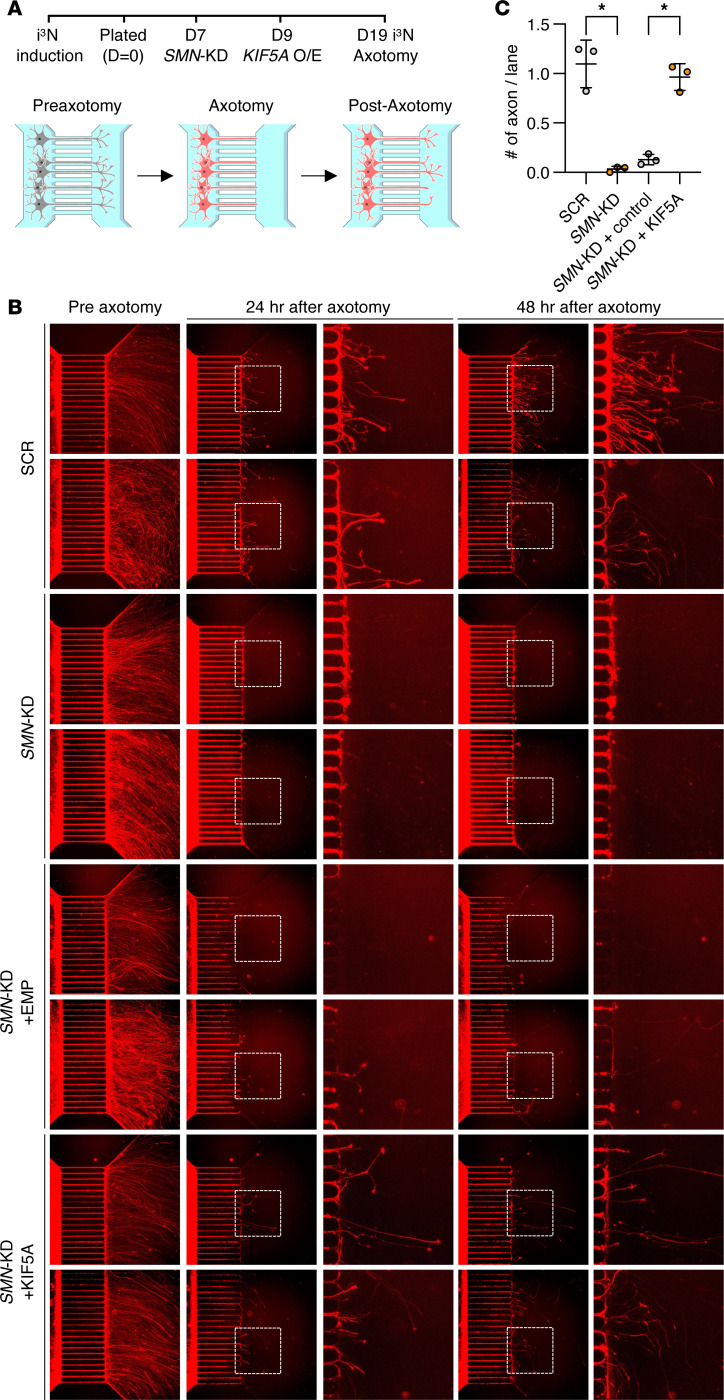
KIF5A overexpression rescues axonal regeneration defects in SMN-deficient neurons. (**A**) Experimental timeline and schema: i^3^Ns underwent *SMN*-KD for 12 days, followed by axonal transection using a microfluidic device. To overexpress KIF5A in SMN-deficient neurons, cells were infected with lentivirus expressing *KIF5A* 2 days after initiating *SMN*-KD. Axonal regeneration was assessed by live-cell imaging at 24 and 48 hours after transection on microfluidic device (XC-450, Xona Microfluidics). (**B**) Representative live-cell images before and after axonal transection. Axons extend from the cell body compartment (left) into the axonal compartment (right). The length of each microchannel is 450 μm. (**C**) Quantification of regenerating axons per microfluidic channel. Data represent the average number of regenerating axons per channel, normalized to control conditions (*n* = 3 images per condition from 2 independent experiments; 17–20 channels per image; >100 channels total). Statistical analysis was performed using 1-way ANOVA followed by Tukey’s multiple comparisons test.

We next investigated the functional effect of KIF5A downregulation in SMA. Given the established role of KIF5A in axonal transport and regeneration (30), we hypothesized that KIF5A downregulation contributes to the axonal growth defects observed in patients with SMA and SMA mouse models (43, 44). We first quantified neurite length and did not find any significant differences in *SMN*-KD i^3^Ns and SMA patient lines (Supplemental Figure 5, A–C). We next examined axonal regeneration in *SMN*-KD i^3^Ns (Figure 4A). *SMN*-KD significantly impaired axonal regeneration compared with control neurons (Figure 4, B and C). KIF5A deficiency alone also reduced axon regeneration (Supplemental Figure 5, D and E) without affecting other kinesin 5 family proteins (Supplemental Figure 5, F and G), consistent with previous reports (30). Importantly, restoring KIF5A expression provided almost full rescue of the axonal regeneration defect in *SMN*-KD i^3^Ns, independent of other protein family members (Figure 4, B and C, and Supplemental Figure 5, H and I). Thus, KIF5A functions downstream of, or parallel to, SMN in the maintenance of axonal integrity.

**Figure 5:**
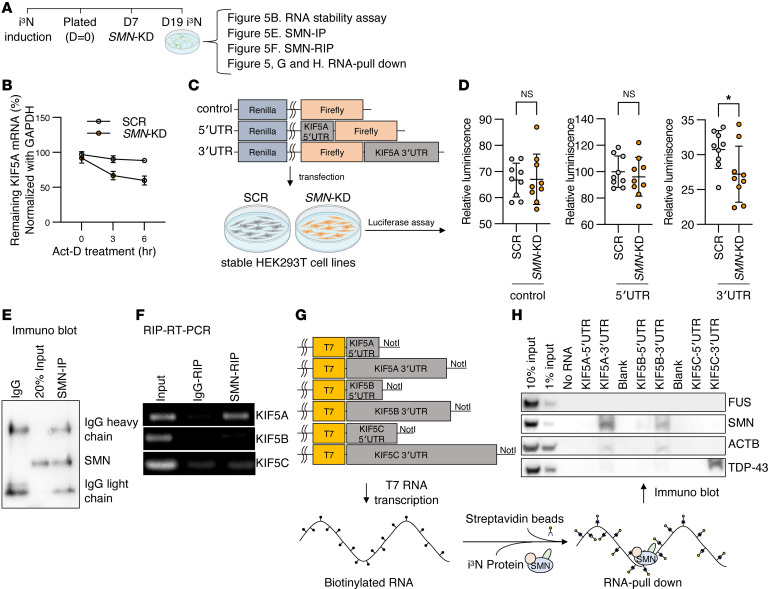
SMN contributes to *KIF5A* mRNA stability through 3′-UTR–dependent mechanism. The relationship between SMN protein levels and KIF5A mRNA stability was investigated. (**A**) Experimental schematic. At 12 days after induction, *SMN* knockdown (*SMN*-KD) and scrambled control (SCR) i^3^Ns were collected for the assays shown in panels **B** and **E**–**H**. (**B**) *SMN*-KD and SCR i^3^Ns were treated with actinomycin D (Act-D, 10 μg/mL) to inhibit de novo RNA synthesis, and cells were harvested at the indicated time points for qPCR analysis. KIF5A mRNA declined more rapidly in *SMN*-KD neurons than in SCR controls, with differences at 3 hours and 6 hours. Statistical significance was determined by 2-way ANOVA followed by Šídák’s multiple comparisons test. *n* = 3 independent experiments. (**C**) Schematic of the dual-luciferase reporter assay used to assess UTR-dependent regulation of KIF5A expression. Luciferase reporters containing no UTR, the KIF5A 5′-UTR, or the KIF5A 3′-UTR were transfected into stable SCR or *SMN*-KD HEK293T cells (Supplemental Figure 6, A–C). (**D**) Quantification of luciferase activity. Reporter activity from control and 5′-UTR constructs was unchanged between SCR and *SMN*-KD cells, whereas the KIF5A 3′-UTR reduced reporter activity in *SMN*-KD cells. (**E**) Immunoprecipitation (IP) using an anti-SMN antibody confirming efficient pulldown of SMN protein from i^3^N lysates. (**F**) RNA immunoprecipitation (RIP) followed by RT-PCR analysis demonstrating preferential enrichment of KIF5A mRNA over KIF5B mRNA in SMN immunoprecipitants. (**G**) Schematic of the RNA pulldown assay using biotinylated 5′ and 3′-UTRs of KIF5A, KIF5B, and KIF5C incubated with i^3^N lysates. (**H**) Immunoblot analysis of RNA pulldown fractions showed recovery of SMN with KIF5A 3′-UTRs and, to a lesser extent, with the KIF5B 3′-UTR, but not with any 5′-UTRs or KIF5C UTRs. FUS was not detected. Beads-only samples without RNA (No RNA) were used as a negative control, and blank lanes contained no sample.

Finally, we investigated the mechanism underlying *KIF5A* mRNA reduction following *SMN*-KD. First, we considered the possibility that SMN deficiency might alter *KIF5A* pre-mRNA splicing. However, reanalysis of our RNA-seq data revealed no clear splicing abnormalities in *KIF5A*. We then hypothesized that SMN deficiency might instead reduce *KIF5A* mRNA stability. To test this, we treated *SMN*-KD i^3^N with actinomycin D to halt new RNA synthesis and measured *KIF5A* mRNA levels over time by qPCR, using *GAPDH* as a stable control. Compared with control samples, *SMN*-KD neurons exhibited an accelerated decline in *KIF5A* mRNA levels, indicating reduced mRNA stability (Figure 5, A and B). We next examined whether this effect is mediated through untranslated regions of KIF5A mRNA. Given the low and variable transfection efficiency of postmitotic neurons, we performed luciferase reporter assays in HEK293T cells as a heterologous system for quantitative assessment of UTR-dependent regulation. Stable *SMN*-KD HEK293T cells were generated by lentiviral transduction followed by puromycin selection (Supplemental Figure 6, A–C), and dual-luciferase reporters containing no UTR, the KIF5A 5′-UTR, or the KIF5A 3′-UTR were transfected (Figure 5C). While luciferase activity from control and 5′-UTR–containing reporters was unchanged between control and *SMN*-KD cells, incorporation of the KIF5A 3′-UTR resulted in a significant reduction of reporter activity in *SMN*-KD cells (Figure 5D). These results indicate that SMN regulates KIF5A expression in a 3′-UTR–dependent manner.

To determine whether SMN interacts with KIF5A mRNA, we performed immunoprecipitation (IP) using an anti-SMN antibody (Figure 5E), followed by RNA extraction (RNA-IP) and RT-PCR analysis from i^3^Ns (Figure 5F). KIF5A mRNA was specifically enriched in SMN-IP samples (Figure 5F), compared with KIF5B or KIF5C, suggesting preferential enrichment of KIF5A transcripts in SMN immunoprecipitates. We further validated this association using RNA pulldown assays with biotinylated 5′- and 3′-UTRs of KIF5A, KIF5B, and KIF5C incubated with i^3^N lysates (Figure 5, A, G, and H). SMN was robustly recovered with KIF5A 3′-UTR and, to a lesser extent, with the KIF5B 3′-UTR, whereas another RNA-binding protein, FUS, was not detected in these pulldown fractions. Together, these experiments indicate that SMN is enriched in complexes containing *KIF5A* mRNA and functionally regulates its expression through a 3′-UTR–dependent mechanism. We note that additional RNA-binding proteins (including ACTB and TDP43) can also be recovered in *KIF5A* 3′-UTR pulldown assays, suggesting that the *KIF5A* 3′-UTR is part of a broader RNP complex rather than establishing direct SMN–RNA binding.

Finally, to test whether specific SMN domains contribute to the association between SMN and *KIF5A* mRNA, we generated FLAG-tagged SMN constructs carrying Tudor domain deletions or point mutations (W102A and Y130A) based on previous work (45), as well as a truncated axonal SMN construct (46), and performed RNA IP (RIP) assays (Supplemental Figure 6, D–G). The truncated axonal SMN construct, which failed to coimmunoprecipitate endogenous SMN, did not associate with *KIF5A* mRNA, whereas Tudor domain–deficient or mutant SMN constructs retained detectable association with *KIF5A* mRNA. Importantly, these Tudor-deficient constructs coimmunoprecipitated endogenous WT SMN, suggesting that the observed association is likely mediated indirectly through endogenous SMN (Supplemental Figure 6, F and G). Thus, while these experiments indicate that SMN associates with *KIF5A* mRNA either directly or indirectly, they do not allow definitive assignment of the Tudor domain as the sole mediator of this interaction. Taken together, these results suggest mechanisms underlying how *KIF5A* mRNA stability is compromised in the absence of SMN.

## Discussion

Recent studies have identified molecular links between SMA and ALS, suggesting shared pathogenic mechanisms involving RNA-binding proteins and spliceosomal dysfunction. For example, interactions between FUS and SMN proteins have been reported, linking ALS and SMA pathogenesis through shared RNA processing pathways (47). Additionally, the ASC-1 complex, implicated in RNA metabolism, has been identified as a molecular link between ALS and SMA (48). Moreover, defects in spliceosome integrity have been observed in both ALS and SMA, highlighting common molecular pathways underlying these motor neuron diseases (49). Our results with KIF5A and SMN now provide an additional link. Our data extend the known role of KIF5A beyond ALS and HSP (24–29), identifying its dysregulation as a novel contributor to SMA pathogenesis, which is supported by a recent study on SMA-model mice (50). Consistent with prior studies in Alzheimer’s disease, Parkinson’s disease, multiple sclerosis, and *SOD1* mutant astrocytes (31–34), these findings suggest that KIF5A downregulation is a common pathogenic feature across diverse neurological disorders.

KIF5A may serve as a critical molecular link connecting distinct motor neuron disorders through common pathways, underscoring its potential as a shared therapeutic target. Notably, prior transcriptomic analysis of laser-capture microdissected motor neurons from SMA model mice did not detect *KIF5A* or *ACAT2* downregulation at early postnatal stages (P2–P5), with changes emerging only at later stages (P10) (37). Thus, in the mouse in vivo context, reduced KIF5A expression may partly reflect disease progression–associated secondary effects, although SMN deficiency likely contributes to KIF5A dysregulation. We note that a previous synaptosome proteomics study in an SMA mouse model did not report reductions in KIF5A (51). Differences in disease stage, subcellular fraction analyzed, and detection sensitivity between proteomics and transcript/protein measurements may contribute to this apparent discrepancy. In contrast, the consistent reduction of KIF5A observed across multiple human iPSC–derived motor neuron models following acute SMN depletion supports a model in which SMN deficiency can directly contribute to KIF5A dysregulation, potentially sensitizing motor neurons to subsequent degeneration.

Axonal integrity is crucial for motor neuron function — it facilitates the transport of essential cargoes necessary for neuronal maintenance, growth, and synaptic function. The considerable distance between motor neuron cell bodies and neuromuscular junctions (NMJs) poses unique challenges, making motor neurons particularly vulnerable to disruptions in axonal transport (3, 52). Previous studies have demonstrated that SMN deficiency impairs axonal elongation (43, 44). KIF5A deficiency results in similar axonal regeneration deficits (30). Taken together, these findings suggest that impaired axonal regeneration may represent a downstream axonal response shared between SMA and ALS. Our findings are consistent with these observations and identify KIF5A as a critical downstream mediator of SMN deficiency, whose restoration significantly improves axonal regeneration. Current SMA treatments primarily focus on restoring SMN protein levels, significantly improving clinical outcomes (11, 12, 16). However, despite their clinical success, existing therapies do not fully address all downstream consequences of SMN deficiency, particularly disruptions in RNA processing, axonal transport, and cytoskeletal integrity that contribute to motor neuron dysfunction and degeneration (3, 18, 19). Thus, combinatorial therapies and SMN-independent approaches are actively being explored to enhance therapeutic efficacy (3). Targeting KIF5A may complement existing therapies by directly addressing axonal transport deficits. Future studies will be required to determine whether restoring KIF5A expression in vivo can mitigate disease phenotypes in SMA animal models.

Strategies to increase KIF5A levels, such as enhancing mRNA stability, direct protein restoration, or pharmacological activation, could enhance the efficacy of SMN-restoring therapies, particularly in patients with severe SMA phenotypes or those initiating treatment later in disease progression. Future studies should explore the therapeutic potential of these or similar approaches in SMA models to determine their efficacy in restoring axonal transport and improving motor neuron function. Future research should also focus on elucidating the precise molecular mechanisms by which KIF5A influences axonal transport, identifying specific cargoes affected by KIF5A downregulation, and determining the temporal dynamics of these transport deficits in SMA. Additionally, it will be important to investigate whether restoring KIF5A expression or function can ameliorate disease phenotypes in vivo using SMA animal models and to explore the potential for small molecule drugs, ASOs, or gene therapies to increase KIF5A expression or activity in SMA motor neurons. Such studies could provide valuable insights into patient stratification and personalized therapeutic approaches.

In conclusion, our findings identify KIF5A as a previously unrecognized downstream mediator of SMN deficiency, highlighting its critical role in SMA pathogenesis and supporting its potential as a novel therapeutic target. Future studies targeting KIF5A may provide opportunities for therapeutic intervention, ultimately improving outcomes for patients with SMA.

## Methods

### Sex as a biological variable.

Both male and female mice were used in this study. Human iPSC lines were derived from donors of both sexes. Because the primary goal of the study was to identify SMN-regulated molecular mechanisms in motor neurons, experiments were not designed to evaluate sex-specific effects.

### Stem cell culture and differentiation into motor/i^3^Ns.

Human iPSCs from both healthy donors and patients with SMA (Supplemental Table 1) were maintained on Matrigel-coated plates in mTeSR plus medium (Supplemental Table 3 for all medium information) and passaged every 4–7 days using ReLeSR (35, 53–55). Motor neuron differentiation was initiated using a modified dual-SMAD inhibition protocol (35). Briefly, iPSCs were dissociated with Accutase and seeded as embryoid bodies (EBs) in ultra-low adhesion plates in iMN basal medium (a 1:1 mixture of Advanced DMEM/F12 and Neurobasal media supplemented with 100 μM ascorbic acid, P/S, Glutamax, NEAA, N2 supplement, and B27 supplement). On day 0, cells were supplemented with 40 μM SB431542, 0.2 μM LDN193189, and 3 μM CHIR99021. On day 2, after initial EB formation, the medium was refreshed with the iMN basal medium supplemented with 100 nM retinoic acid and 500 nM smoothened agonist. Subsequent media changes on days 4, 7, and 9 included additional factors such as BDNF, GDNF, and DAPT (γ-secretase inhibitor) (10 μM), and to promote motor neuron specification and maturation. On day 10, EBs were dissociated using papain-dissociation system and replated onto poly-D-lysine/laminin-coated plates in an iMN basal medium supplemented with BDNF, GDNF, and DAPT. Media were subsequently half-changed every 2–3 days with iMN basal medium supplemented with BDNF and GDNF. For the generation of i^3^N, hES Cell; H1 were used. The differentiation of hESCs into neurons was achieved by inducing the overexpression of NGN2 as previously described (54).

### FACS.

Cells were dissociated into single-cell suspensions using a Papain dissociation system and filtered to remove aggregates. Cells were resuspended in sorting buffer consisting of Neurobasal medium (phenol free) supplemented with N2, B27, and ROCK inhibitor. Sorting was performed using a BD FACS Aria III cell sorter (BD Biosciences) equipped with a 100 μm nozzle. Singlet cells were gated based on forward scatter (FSC) and side scatter (SSC) parameters (FSC-A versus FSC-H, SSC-A versus SSC-H) to exclude doublets and aggregates. Cells positive for Td-Tomato were sorted into collection tubes containing culture medium with ROCK-inhibitor and kept on ice until plating.

### shRNA cloning, lentiviral packaging, and cellular transduction.

shRNA sequences targeting *SMN1* and *2* (ATCTGTGAAGTAGCTAATAAT) and a scrambled nontargeting control (GATATCGCTTCTACTAGTAAG) were obtained from the Broad Institute Genetic Perturbation Platform (GPP) portal. Complementary oligonucleotides containing 4-nucleotide overhangs were synthesized, annealed, and ligated into the pRSITER-U6Tet-(sh)-EF1-TetRep-2A-TagRFP vector (Cellecta). Ligated plasmids were transformed into Stbl3 competent cells (Thermo Scientific, C737303) and cultured at 30°C. Large-scale plasmid DNA was prepared using a Qiagen Maxiprep kit and used for lentiviral production. Second-generation packaging plasmids psPAX2 and pMD2.G were cotransfected with shRNA constructs into Lenti-X 293T cells (Takara) using Lipofectamine 2000 (Invitrogen). Lentiviral packaging was performed at the Stanford Gene Vector and Virus Core (GVVC). Viral supernatants were collected at 48 and 72 hours after transfection and concentrated by ultracentrifugation (50,000*g* for 2 hours at 4°C). Viral titers were determined by serial dilution followed by fluorescence-based quantification of RFP^+^ cells. Target i^3^N and iMN cells were infected at day 3 after plating (D = 3) at a multiplicity of infection (MOI) of 1. To induce shRNA expression, doxycycline was added to the culture medium. While the standard concentration is 2 μg/mL, a reduced concentration of 0.5 μg/mL was used to achieve mild KD (Supplemental Figure 1, A–C). To generate stable *SMN*-KD HEK293T cells, an independent MISSION shRNA targeting the SMN 3′-UTR (TRCN0000359379; target sequence: GGGTAACTCTTCTTGATTAAA) was used. Cells were transduced with lentivirus at a MOI of 2, and MISSION pLKO.1-puro nontarget shRNA was used as the control. Lentiviral packaging and titration, followed by puromycin selection, were performed at the Stanford GVVC. Following transduction, cells were selected and expanded for downstream analyses.

For *KIF5A*-KD and overexpression experiments, lentiviral vectors were obtained from VectorBuilder. The shRNA lentivirus targeting human KIF5A (shKIF5A) was generated using the pLV[shRNA]-mCherry-U6>hKIF5A (shRNA#1) vector (Vector ID: VB231128-1302rda; target sequence: GCGTTGTGAGCTTCCTAAATT). The corresponding scrambled control virus was produced using the pLV (shRNA)-mCherry-U6>Scramble_shRNA#1 vector (Vector ID: VB010000-0002vzc). For KIF5A overexpression, recombinant lentivirus was generated using the pLV[Exp]-EF1A>hKIF5A (NM_004984.4) vector (Vector ID: VB250107-1212agt), with pLV[Exp]-EGFP/Puro-EF1A>mCherry (Vector ID: VB010000-9492agg) used as a control. All lentiviruses were formulated in HBSS buffer and stored at –80°C.

### SMN restoration treatments in iPSC-derived motor neurons.

To evaluate the effects of SMN restoration in SMA patient–derived motor neurons, we performed 2 independent treatment strategies: lentiviral SMN overexpression and nusinersen administration. For lentiviral SMN overexpression, we obtained pLV[Exp]-EGFP-EF1A>hSMN1[NM_000344.4] (Vector ID: VB900104-4718uje) from VectorBuilder. As an empty control, an EGFP-only lentivirus was used. On day 10 after plating, lentivirus was added at a MOI of 2, and cells were cultured for an additional 10 days before sample collection. For nusinersen treatment, we used a chemically synthesized ASO obtained from MedChem Express (HY-112980, Chemical Abstracts Service [CAS] no. 1258984-36-9). A nontargeting ASO control was obtained from Qiagen (Antisense LNA GapmeR Standard, 339511). On day 10 after plating, nusinersen and nontargeting ASO was added to the culture medium at a final concentration of 2 μM. The medium was left unchanged for 2 days, after which half-medium changes were performed every 2–3 days for 20 days before sample collection.

### Generation of stable SMN-KD HEK293T cells.

Stable *SMN*-KD HEK293T cells were generated by lentiviral transduction using MISSION shRNA targeting the SMN 3′-UTR or a scrambled nontargeting control. HEK293T cells were transduced at a multiplicity of infection (MOI) of 2. After 1 passage, cells were subjected to selection with puromycin (1 μg/mL) and passaged once more. Cells were expanded and used for downstream experiments once they reached confluency. Stable SCR and *SMN*-KD cells were used for luciferase assays (Figure 5, C and D) and transfection-based experiments (Supplemental Figure 6).

### Dual-luciferase reporter assay.

For luciferase reporter assays, the pmirGLO Dual-Luciferase miRNA Target Expression Vector (Promega, E1330) was used as the backbone. Human KIF5A 3′-UTR or 5′-UTR sequences were cloned down- or upstream of the firefly luciferase gene. SCR or *SMN*-KD HEK293T cells were seeded at 1.5 × 10^5^ cells per well in poly-D-lysine–coated 12-well plates for 3 biological replicates per condition. Cells were transfected with luciferase reporter constructs using ViaFect Transfection Reagent (Promega) according to the manufacturer’s instructions. Forty-eight hours after transfection, luciferase activity was measured using a dual-luciferase assay system, and firefly luciferase activity was normalized to Renilla luciferase activity using Nano-Glo Dual-Luciferase Reporter Assay System (Promega E2920).

### SMN truncation and mutant constructs.

A series of SMN truncation and mutant constructs was generated by GenScript by cloning the corresponding SMN cDNA fragments into the pCMV-3Tag-1a expression vector. All constructs were sequence-verified prior to use. For RIP experiments, stable *SMN*-KD HEK293T cells were seeded at 3 × 10^5^ cells per well in poly-D-lysine–coated 6-well plates. Cells were transfected with the indicated SMN expression constructs using Lipofectamine 3000. Seventy-two hours after transfection, cells were harvested in IP buffer and subjected to IP followed by RNA extraction for RIP analyses.

### Immunoblotting.

Cells were lysed on ice in either ice-cold RIPA buffer (Sigma-Aldrich, R0278) supplemented with a protease/phosphatase inhibitor cocktail (Cell Signaling Technology, 5872S) for general Western blotting, or in IP buffer consisting of 25 mM HEPES-NaOH (pH 7.4), 150 mM NaCl, 5 mM MgCl_2_, 0.5 mM DTT, and 1% NP-40. For IP, the buffer was additionally supplemented with EDTA-free protease inhibitors (Roche) and RNase inhibitors (Takara). Lysates were incubated at 4°C for 10 minutes with gentle mixing. The lysates were then centrifuged at 20,000*g* for 10 minutes at 4°C to remove cellular debris, and the resulting supernatant was utilized for BCA protein assay (Invitrogen, 23225) to quantify protein concentrations. Unless otherwise specified, 5–10 μg of protein from each sample was denatured in LDS sample buffer (Thermo Scientific, B0007) containing 10% 2-mercaptoethanol (Sigma-Aldrich) at 80°C for 10 minutes. The denatured samples were subjected to electrophoresis on 4%–12% Bis-Tris Plus Gels (Thermo Fisher, NW04127BOX) and subsequently transferred onto 0.45 μm nitrocellulose membranes (Bio-Rad, 162-0115) using a semidry transfer method (Trans-Blot Turbo Transfer System). The membranes were blocked using EveryBlot Blocking Buffer (Bio-Rad, 12010020) or 5% nonfat dry milk in TBS-T for 1 hour, followed by overnight incubation at 4°C with specific antibodies against the target proteins (Supplemental Table 4). After triple washing, the membranes were incubated with horseradish peroxidase–conjugated (HRP-conjugated) secondary antibodies (anti-mouse IgG or anti-rabbit IgG, both at 1:5,000 dilution) for 1 hour. Detection of protein bands was performed using the Amersham ECL Prime kit (Cytiva, RPN2232), and images were captured using the ChemiDoc XRS+ System (Bio-Rad). Band intensities were quantified using Fiji.

### Immunofluorescence.

Cells were fixed with 4% formaldehyde (Electron Microscopy Sciences 15710) in PBS (–) at room temperature for 15 minutes, followed by 4 washes with PBS (–). They were then permeabilized with 0.2% Triton X-100 in blocking buffer (5% normal goat serum in PBS [–]) at room temperature for 1 hour. After permeabilization and blocking, cells were incubated overnight at 4°C in blocking buffer containing specific antibodies (Supplemental Table 4). Following primary antibody staining, cells were washed 4 times with 1× PBS and then incubated with secondary antibodies in blocking buffer containing Hoechst 33342 (1:1,000) at room temperature for 1 hour. After this incubation, cells were washed 4 times with PBS (–) and imaged using a Leica DMI6000 B microscope equipped with a 20× objective and a Hamamatsu ORCA-flash 4.0 camera.

### smFISH.

Custom Stellaris FISH probes targeting human KIF5A were designed by Stellaris (LGC Biosearch Technologies). Control and *SMN*-KD i^3^Ns at 20 days postplating (DPP) were hybridized with the KIF5A Stellaris probe set labeled with Quasar 670 (MF-1065-5, LGC Biosearch Technologies) according to the manufacturer’s instructions. Images were acquired using a Nikon Crest spinning-disk confocal microscope and analyzed using ImageJ software (NIH).

### Total RNA extraction.

Total RNA was isolated using RNeasy mini kit (Qiagen) combined with DNase I treatment according to the manufacturer’s protocol.

### qPCR.

Total RNA (200 ng) was reverse transcribed into cDNA using the High Capacity cDNA Reverse Transcription Kit (Thermo Fisher, 43-688-13). Quantitative PCR (qPCR) was performed using the PowerUp SYBR Green Master Mix kit and detected with the QuantStudio 3 Real-Time PCR System (Thermo Fisher). The primers used are listed in Supplemental Table 5.

### Total RNA-seq.

Total RNA from cells treated with scrambled shRNA or *SMN1/2*-targeting shRNA was used to construct RNA-seq libraries using a ribosomal RNA depletion protocol (RiboErase), performed by the Stanford Genomics Core Facility. The resulting libraries were quantified, pooled, and sequenced on an Illumina NovaSeq 6000 using an S2 flow cell with 150 bp paired-end reads.

### Gene expression and splicing analysis.

Adapter sequences in FASTQ files were trimmed using fastp. The trimmed FASTQ files were then used for transcript quantification with Salmon, followed by differential gene expression analysis using DESeq2, as previously described (56).

For splicing analysis, the adapter-trimmed FASTQ files were aligned to the human genome (hg38) according to ENCODE’s recommended settings using STAR. The uniquely mapped, properly paired reads were subsequently analyzed for splicing events using LeafCutter, which identified cryptic splicing occurrences (57).

### RIP.

RIP was performed using an anti-SMN antibody and Protein G Dynabeads (Thermo Fisher Scientific, 10004D). RNA was extracted from the immunoprecipitates using TRIzol reagent (Thermo Fisher Scientific, 15596018), and 200 ng of RNA was reverse transcribed into cDNA using the High-Capacity cDNA Reverse Transcription Kit (Thermo Fisher Scientific, 4368813). cDNA was analyzed by to reverse transcription PCR (RT-PCR) using primers specific for *GAPDH*, *KIF5A*, *KIF5B*, and *KIF5*C (see Supplemental Table 5). PCR products were separated on a 2% agarose gel at 140 V for 20 minutes and visualized using the ChemiDoc XRS+ System (Bio-Rad).

### RNA pulldown assay.

To generate KIF5A, KIF5B, and KIF5C 3′- and 5′-UTR RNA probes, each UTR sequence was cloned into the pcDNA3.1(+) vector by GenScript. Plasmids were linearized using NotI and purified with a DNA cleanup kit (Zymo Research). In vitro transcription was performed using T7 RNA polymerase (Takara) in the presence of a biotin RNA labeling mix (Roche) and RNase inhibitor (Takara). The resulting biotinylated RNAs were purified with an RNA cleanup kit (Zymo Research) following the manufacturer’s protocol. For the pulldown, IP buffer lysates from i^3^Ns at 20 DPP (300 μg total protein) were prepared as described above and incubated with 3 μg of biotinylated RNA for 1 hour at room temperature. Streptavidin Dynabeads (Invitrogen) were then added and incubated for an additional 1 hour at 4°C. Beads were washed 3 times with IP buffer containing RNase inhibitor, boiled in 4× Laemmli buffer with 10% 2-ME for 5 minutes at 95°C, and analyzed by Western blotting.

### RNA stability (actinomycin D) assay.

SCR or *SMN*-KD i^3^N cells were exposed to actinomycin D (10 μg/mL) to inhibit RNA transcription. RNA was extracted at various time points (0, 3, and 6 hours) and analyzed by qPCR to assess the stability of *KIF5A* mRNA.

### Axon regeneration assay.

i^3^N cells were plated at 5 × 10^4^ cells per PDL/Laminin-coated microfluidic device (XC450, Xona Microfluidics). Axons were transected by washing twice with RIPA buffer (10 seconds each), followed by 3 washes each with PBS (–) and i^3^N medium containing 3.3 μg/mL iMatrix-511 (PeproTech, RL511S). Axonal regeneration was quantified by measuring the number and length of regenerating axons at 24 and 48 hours after transection. Images were acquired using a Leica DMI6000 B microscope equipped with a 10 × objective and a Hamamatsu ORCA-Flash 4.0 camera. For measuring axon length during recovery, axons were counted from a point 5 μm away from the injury site (at the end of the microchannels).

### High-content imaging and analysis.

For confirmation of differentiation efficiency (Supplemental Figure 3), immunocytochemistry-processed (ICC-processed) cells were imaged from 5 fields per well for day 30 (D30) neurons using an ImageXpress Micro Confocal system (Molecular Devices) with Neuronal Profiling software (version 4). Cells were labeled with Hoechst (nuclei), Alexa Fluor 488 (ISL1/2), Alexa Fluor 555 (HB9), and Alexa Fluor 647 (βIII-tubulin/TUJ1). Imaging was performed using the following filter sets (excitation/emission): nuclei, broad blue (365/535); ISL1/2, green (475/535); HB9, red (555/595); and βIII-tubulin, far red (630/695). For quantification of βIII-tubulin–positive neurons, image analysis was performed using Neuronal Profiling (version 4). Intact nuclei were first identified based on Hoechst staining and defined as traced nuclei with typical intensity levels below the threshold brightness of pyknotic cells. Each traced nuclear region was then expanded by 50% to define a perinuclear area and cross-referenced with βIII-tubulin staining to identify neurons. The number of nuclei was subsequently quantified based on the presence or absence of ISL1/2 and/or HB9 nuclear staining within βIII-tubulin–positive cells. For neurite length analysis (Supplemental Figure 5, A–C), TUJ1 images acquired under identical imaging settings were used. Neurites were traced, and total neurite length per cell (μm/cell) was quantified using MetaXpress software (Molecular Devices).

### Primary mouse cortical neurons.

Mice were bred and used as approved by the Administrative Panel of Laboratory Animal Care (APLAC) of Stanford University, an institution accredited by the Association for the Assessment and Accreditation of Laboratory Animal Care (AAALAC). Primary mouse cortical neurons were dissociated into single-cell suspensions from E16.5 mouse cortices using a papain dissociation system, as previously described (58). Neurons were seeded onto PDL-coated plates and grown in Neurobasal medium supplemented with B-27, GlutaMAX, and penicillin-streptomycin (Supplemental Table 3). Half-media changes were performed every 4–5 days. Neurons were plated in 24-well plates (350,000 cells/well). Three days after plating, primary neurons were treated for 3 days with 100 nM-siRNA targeting *Smn* (Dharmacon, L-044280-00) or a nontargeting control (Dharmacon, D-001810-10) using DharmaFECT 3 (Dharmacon, T-2003) in Opti-MEM buffer.

### Mouse IHC analysis.

For SMNΔ7 model mice (The Jackson Laboratory), P10 mice and age-matched controls were euthanized, and spinal cords were collected. Mice were anesthetized and perfused transcardially with phosphate-buffered saline (PBS). Spinal cords were then carefully dissected and rinsed in ice-cold PBS. The spinal cords were fixed with 4% PFA in PBS at 4°C for 48 hours and then stored in 30% sucrose in PBS. Fixed spinal cords were mounted in OCT and dissected into 50 μm–thick sections using a Leica CM3050 S Cryostat with a cryo-microtome (Leica) and mounted on slides, which were stored at –80°C until used for IHC. For IHC, each sample was washed twice with PBS (–), and blocked with 5% NGS, 1% BSA, and 0.4% Triton-X in PBS (–) for 1 hour at room temperature. The first antibody (Supplemental Table 4) was applied and incubated at 4°C overnight. After incubation with the first antibody, samples were washed 3 times with PBS (–) and stained with the second antibody for 1 hour. They were then washed 3 times with PBS (–), mounted with ProLong Gold (Thermo Fisher Scientific, P36931) overnight, and captured using a microscope. All images were taken under the same conditions and analyzed with Fiji. For analysis, motor neurons were identified using ChAT staining. By applying a threshold, the ChAT-positive area was recognized as the motor neuron area (region of interest, ROI). After analyzing ChAT intensity, the same ROI was applied for SMN and KIF5A staining, and each intensity was calculated.

For analysis of the milder SMA model, *Smn^2B/–^* mice carrying a Cre-inducible Sun1–sfGFP reporter and the ChAT-Cre driver were used to label motor neuron nuclei resulting in reliable GFP labeling of motor neuron nuclear envelopes in the spinal cord. These *Smn^2B/–^* ChAT-Cre Sun1–sfGFP mice were generated through extensive breeding at Jackson Laboratory. P17 and littermate control mice were anesthetized and perfused transcardially with 4% PFA. Mice were submersion fixed with 4% PFA in PBS at 4°C for 24 hours. Spinal cords were then dissected and stored in 30% sucrose in PBS for 3 days before mounting in OCT. Lower lumbar spinal segments were sectioned into 30 μm–thick, free-floating sections in 0.3% PBST with 5% NGS for 1 hour at room temperature. The primary antibodies (SMI32 1:1,000, KIF5A 1:100, GFP 1:500) were applied and incubated at 4°C overnight. After incubation with the first antibody, samples were washed 3 times with PBS (–) and stained with the secondary antibodies (all 1:200) overnight at 4°C. They were then washed 3 times with PBS (–) and mounted with ProLong glass antifade mounting media (Thermo Fisher Scientific, P36981). All images were taken on a Zeiss AxioImager.Z1 microscope equipped with an Apotome under the same conditions and analyzed with Fiji. For analysis, motor neurons were identified using Sun1-sfGFP fluorescence. SMI32 was used to indicate the region of interest (i.e., the motor neuron cell body) and average KIF5A intensity was measured as an average within the region of interest.

### Statistics.

Statistical analyses were performed using GraphPad Prism (version 10.4.1). Data are presented as mean ± SD unless otherwise indicated. For comparisons involving multiple groups, Šídák’s multiple-comparison test was used as appropriate. Statistical analyses were performed as described in the figure legends. For experiments involving SMN overexpression, where large fold changes were observed, individual qPCR and immunoblot quantification values were log_2_ transformed prior to statistical analysis (Figure 3, D and F). For 2-group comparisons, an unpaired 2-tailed *t* test with Welch’s correction was used, and for comparisons involving more than 2 groups, 1-way ANOVA followed by Tukey’s multiple comparisons test was applied, unless otherwise indicated. Data distribution was assessed by visual inspection of the data; formal tests of normality were not performed. *P* < 0.05 was considered significant.

### Study approval.

All experiments involving human iPSCs and ESCs were conducted in accordance with Stanford University’s Stem Cell Research Oversight (SCRO) guidelines and approved under protocol SCRO-858. All recombinant DNA work, including vector cloning, lentiviral packaging, and bacterial manipulations, was approved by the Stanford Administrative Panel on Biosafety (APB) under protocol no. APB-5676. Mouse experiments are approved by the APLAC of Stanford University, an institution accredited by the AAALAC.

### Data availability.

The sequencing data generated in this study have been deposited in the Gene Expression Omnibus (GEO) database under accession no. GSE302774. Supporting data values underlying the figures in this manuscript are provided in the Supporting Data Values file. No custom code was generated for this study. All other data supporting the findings of this study are available from the corresponding authors upon reasonable request.

## Author contributions

TA and ADG conceived and supervised the study and wrote the manuscript. TA designed and performed the experiments and analyzed data. YZ and CG performed RNA-seq data analysis. OG, LK, and EM prepared mouse tissue samples for analysis. JSB prepared primary mouse neurons. OS generated and maintained i^3^Ns. HL conducted luciferase assay. JPR analyzed public RNA-seq datasets. PH established the iMN induction system. LZ and CS assisted with ICC and Western blot experiments. CJS, MM, and JWD supervised parts of the project and contributed to experimental design and interpretation.

## Conflict of interest

The authors have declared that no conflict of interest exists.

## Funding support

This work is the result of NIH funding, in whole or in part, and is subject to the NIH Public Access Policy. Through acceptance of this federal funding, the NIH has been given a right to make the work publicly available in PubMed Central.

- 2T32AG047126-06A1 and a fellowship from the Takeda Science Foundation (TA)
- Postdoctoral scholar award from The Phil and Penny Knight Initiative for Brain Resilience at the Wu Tsai Neurosciences Institute, Stanford University, and a fellowship grant from the Larry L. Hillblom Foundation (YZ)
- Milton Safenowitz Postdoctoral Fellowship Program (CG)
- ALS Scholars in Therapeutics award from the Sean M. Healey & AMG Center for ALS, ALS Finding a Cure, and FightMND (JPR)
- Postdoctoral scholar award from The Phil and Penny Knight Initiative for Brain Resilience (HL)
- NIH grants R35NS122306 and F32NS138246, Cure SMA, and the SMA Foundation (EM and CJS)
- SMA Foundation (JWD)
- SMA Foundation (MM)
- NIH grants R35NS137159, U54NS123743, and R01AG064690, Target ALS, The Phil and Penny Knight Initiative for Brain Resilience at the Wu Tsai Neurosciences Institute, Stanford University, and the SMA Foundation (ADG)

## Supplementary Material

Supplemental data

Unedited blot and gel images

Supplemental tables 1-5

Supporting data values

## Version 1 03/26/2026

In-Press Preview

## Version 2 05/22/2026

Electronic publication
