# Supplementary material for: KIF5A downregulation in spinal muscular atrophy links axonal regeneration defects with ALS
Source: JCI Insight. 2026 Mar 26;11(10):e197941. doi: 10.1172/jci.insight.197941 (PMC13232735; doi:10.1172/jci.insight.197941)
Supplement: Supplemental data [file jciinsight-11-197941-s233.pdf]

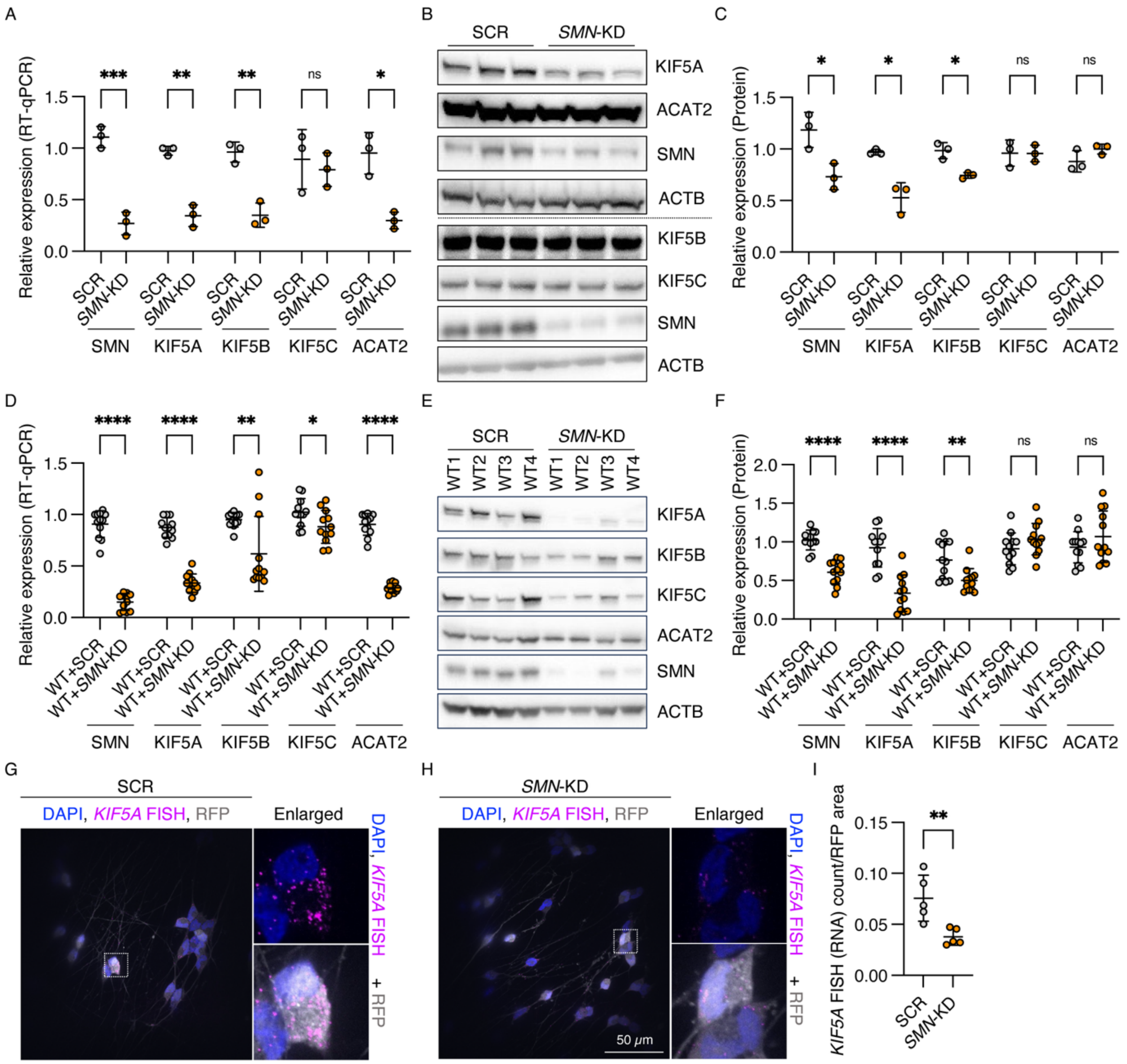

Supplemental Figure 1. ***SMN*** **knockdown reduces KIF5A expression in human neurons**

(**A**) RT-qPCR analysis of i^{3}Neurons (i^{3}Ns) following *SMN* knockdown (*SMN*-KD) showing mRNA levels of kinesin-1 family members (KIF5A, KIF5B, and KIF5C) and ACAT2. Both KIF5A and ACAT2 mRNA levels were reduced compared with SCR i^{3}N. (**B**) Immunoblot analysis of i^{3}Ns lysates following *SMN*-KD showing reduced SMN and KIF5A protein levels, whereas KIF5B, KIF5C, and ACAT2 protein levels remained unchanged. To achieve a mild KD, doxycycline was used at 0.5 μg/ml (standard concentration = 2 μg/ml). Dashed lines indicate that lanes were derived

from different membranes. (**C**) Quantification of immunoblot data shown in (**B**). SMN and KIF5A protein levels were significantly reduced in *SMN*-KD i^3Ns relative to SCR controls, whereas ACAT2 protein levels were not altered. Data represent three independent experiments. Statistical analysis was performed using an unpaired two-tailed t-test with Welch's correction. (**D**–**F**) *SMN*-KD preferentially reduces KIF5A expression in healthy donor–derived motor neurons (iMNs). Motor neurons differentiated from four healthy donor iPSC lines were analyzed (see Supplemental Table 1 for cell line details). (**D**) RT-qPCR analysis of iMNs following *SMN*-KD showing a preferential reduction of KIF5A mRNA compared with other kinesin-1 family members. (**E** and **F**) Immunoblot analysis and quantification of protein levels in iMNs following *SMN*-KD, demonstrating limited or context-dependent changes compared with the robust reduction observed for KIF5A. (**G**–**I**) Single-molecule fluorescence in situ hybridization (smFISH) analysis of KIF5A mRNA in SCR and *SMN*-KD i^3Ns. Representative images from SCR i^3Ns (**G**) and *SMN*-KD i^3Ns (**H**), and quantification of KIF5A mRNA puncta per cell (**I**) demonstrate a reduction of KIF5A transcripts following *SMN*-KD. Statistical analysis was performed using an unpaired two-tailed t-test with Welch's correction (n = 5).

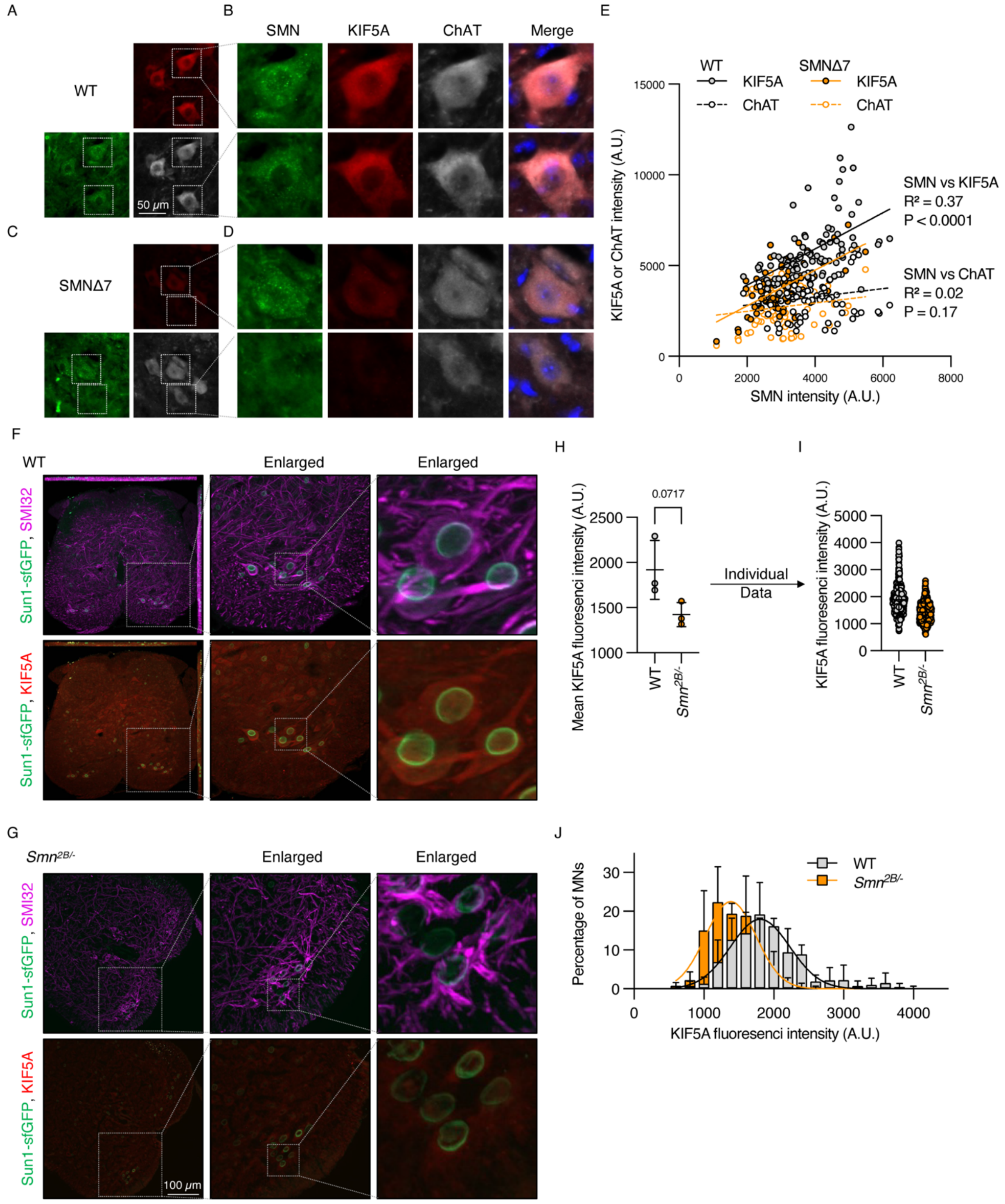

Supplemental Figure 2. KIF5A expression correlates with SMN levels in spinal motor neurons of SMA mouse models

(**A–D**) Representative immunofluorescence images of lumbar spinal cord sections stained for SMN, KIF5A, and the motor neuron marker ChAT from postnatal day 10 (P10) age-matched control mice (**A** and **B**) and SMNΔ7 mice (**C** and **D**). Spinal motor neurons in SMNΔ7 mice exhibited reduced KIF5A expression compared with controls. (**B**) Higher-magnification images of representative motor neurons from control mice indicated in (**A**). (**D**) Higher-magnification images of representative motor neurons from SMNΔ7 mice indicated in (**C**), including examples retaining detectable SMN and KIF5A expression. (**E**) Correlation analysis between SMN and KIF5A fluorescence intensities in individual motor neurons from SMNΔ7 mice, revealing a positive correlation. No such correlation was observed between ChAT and SMN intensities. (**F–J**) KIF5A expression in a milder SMA mouse model. (**F** and **G**) Representative immunofluorescence images of lumbar spinal motor neurons from postnatal day 17 (P17) $Smn^{2B/-}$ mice (**G**) and corresponding control mice (**F**). Motor neuron nuclei were genetically labeled with Sun1–sfGFP (green). Neuronal identity was confirmed by SMI32 immunostaining (Top panel, purple), and KIF5A expression was visualized by immunostaining (bottom panel, red). (**H**) Quantification of KIF5A fluorescence intensity in spinal motor neurons from $Smn^{2B/-}$ mice, showing a trend toward reduced KIF5A expression compared with controls. (**I**) Individual motor neuron KIF5A intensity values corresponding to the quantification shown in (**H**). (**J**) Frequency distribution analysis of KIF5A fluorescence intensity across both SMA mouse models, showing a shift toward lower KIF5A intensity values in $Smn^{2B/-}$ mice.

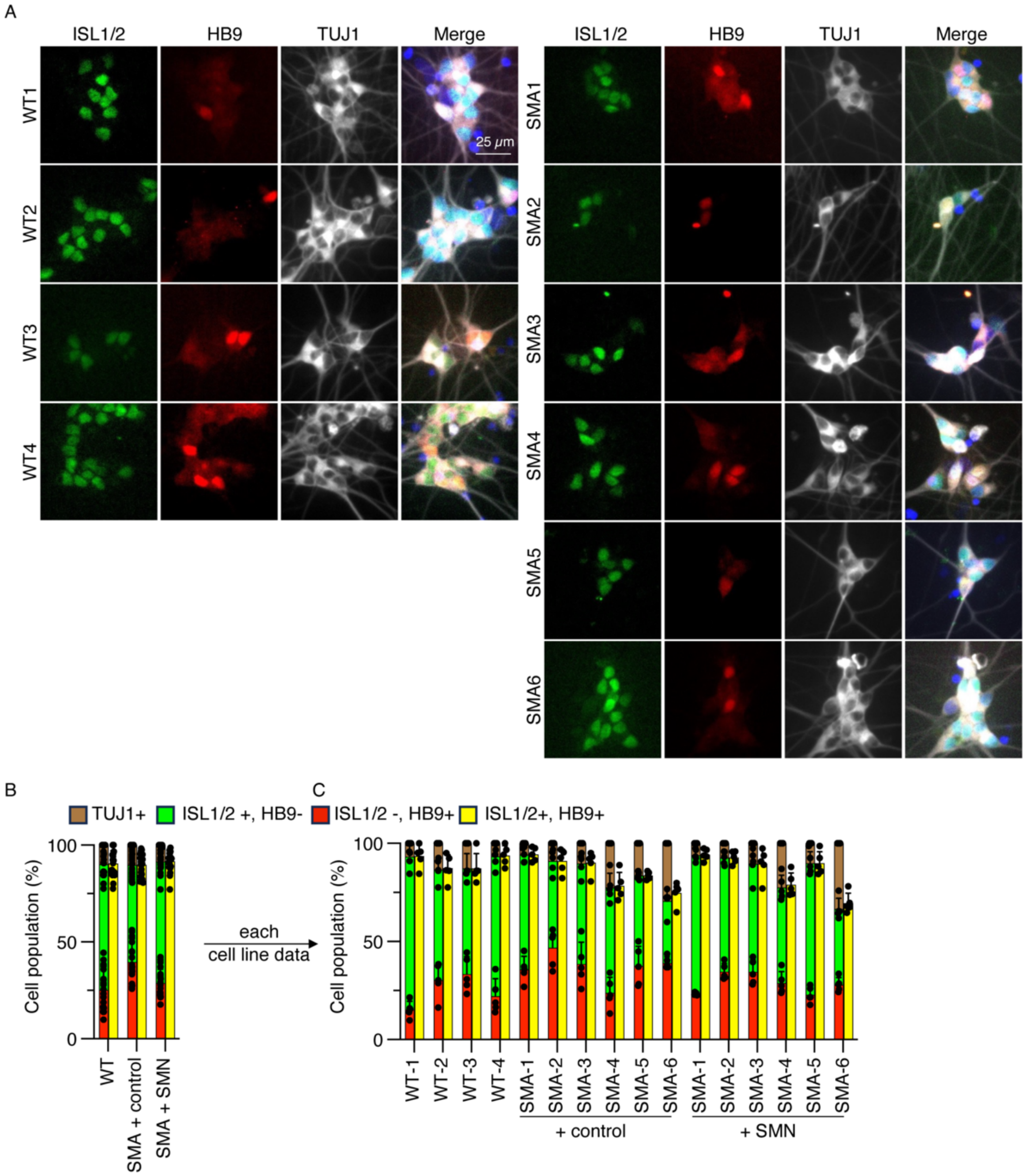

Supplemental Figure 3. Motor neuron differentiation efficiency is consistent across all iPSC lines.

To confirm comparable motor neuron differentiation efficiency between wild-type (WT) and SMA iPSC lines, immunostaining was performed at day 20 of differentiation. (**A**) Representative immunofluorescence images of iPSC-derived motor neurons at day 20 stained for βIII-tubulin (TUJ1), HB9 (red), and ISL1/2 (green), both of which are motor neuron–specific markers. (**B** and

C) Quantification of motor neuron induction efficiency using the ImageXpress system. Five regions per sample were imaged, and five biologically independent samples were analyzed. (**B**) Group-averaged data are shown for WT, SMA + control, and SMA + SMN conditions. (**C**) Data from individual iPSC lines are shown. In the bar graphs, brown indicates the total population of TUJ1-positive nuclei (set to 100%). Red and green indicate nuclei positive for HB9 or ISL1/2 alone, respectively, while yellow represents nuclei positive for either HB9 or ISL1/2. Statistical analysis was performed using one-way ANOVA on group-aggregated values (WT, SMA + control, and SMA + SMN) for TUJ1-positive, HB9-positive, ISL1/2-positive, and HB9+ISL1/2–positive nuclei. No significant differences were observed among groups, indicating comparable motor neuron differentiation efficiency across all iPSC lines.

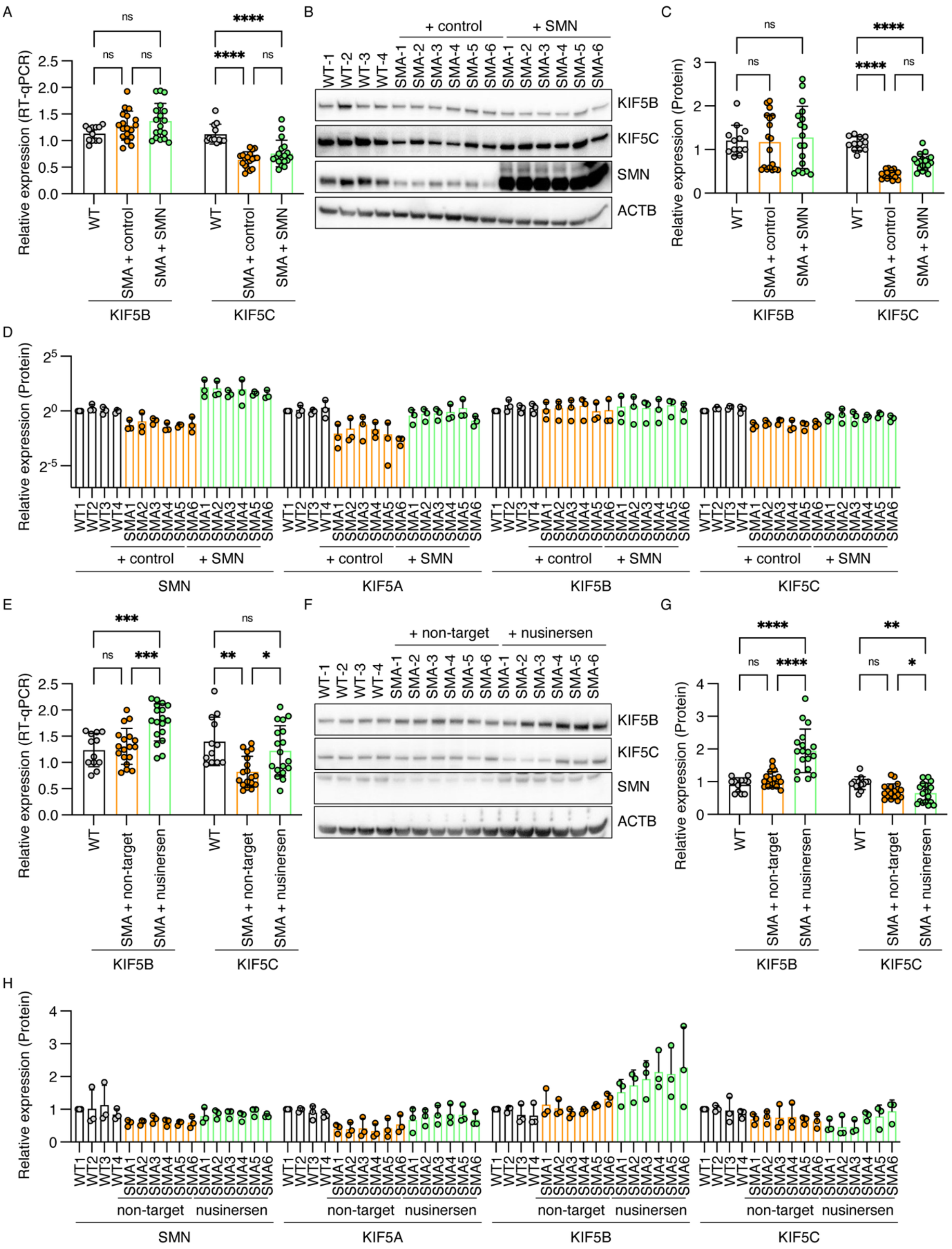

A
Relative expression (RT-qPCR)
ns
****
WT
SMA + control
SMA + SMN
KIF5B
KIF5C
B
+ control
+ SMN
WT-1 WT-2 WT-3 WT-4 SMA-1 SMA-2 SMA-3 SMA-4 SMA-5 SMA-6 SMA-1 SMA-2 SMA-3 SMA-4 SMA-5 SMA-6
KIF5B
KIF5C
SMN
ACTB
C
Relative expression (Protein)
D
Relative expression (Protein)
WT1 WT2 WT3 WT4 SMA1 SMA2 SMA3 SMA4 SMA5 SMA6 SMA1 SMA2 SMA3 SMA4 SMA5 SMA6
+ control
+ SMN
SMN
KIF5A
KIF5B
KIF5C
E
Relative expression (RT-qPCR)
***
**
*
ns
WT
SMA + non-target
SMA + nusinersen
KIF5B
KIF5C
F
+ non-target
+ nusinersen
WT-1 WT-2 WT-3 WT-4 SMA-1 SMA-2 SMA-3 SMA-4 SMA-5 SMA-6 SMA-1 SMA-2 SMA-3 SMA-4 SMA-5 SMA-6
KIF5B
KIF5C
SMN
ACTB
G
Relative expression (Protein)
H
Relative expression (Protein)
non-target
nusinersen
SMN
KIF5A
KIF5B
KIF5C

Supplemental Figure 4. Expression of kinesin-1 family members following SMN restoration in SMA patient-derived motor neurons

(**A**–**D**) Analysis of kinesin-1 family expression following SMN overexpression in SMA patient–derived motor neurons (iMNs). (**A**) RT-qPCR analysis of KIF5B and KIF5C mRNA levels in WT, SMA + control lentivirus, and SMA + SMN overexpression conditions. (**B**) Representative immunoblots showing protein levels of KIF5B and KIF5C under the same conditions. SMN and ACTB bands from Figure 3**E** are shown from the same membrane. (**C**) Quantification of immunoblot data shown in (**B**), indicating limited or context-dependent effects of SMN overexpression on KIF5B and KIF5C protein levels, relative to the restoration of KIF5A shown in Figure 3. (**D**) Individual immunoblot data for each cell line, showing protein levels of SMN, KIF5A, KIF5B, and KIF5C under WT, SMA + control, and SMA + SMN overexpression conditions. These data correspond to the group-averaged quantifications shown in Figure 3**F** and Supplemental Figure 4**C**. (**E**–**H**) Analysis of kinesin-1 family expression following nusinersen treatment in SMA patient-derived iMNs. (**E**) RT-qPCR analysis of KIF5B and KIF5C mRNA levels in WT, SMA + non-targeting ASO, and SMA + nusinersen conditions. (**F**) Representative immunoblots showing protein levels of KIF5B and KIF5C following ASO treatment. (**G**) Quantification of immunoblot data shown in (**F**), indicating no consistent restoration of KIF5B or KIF5C protein levels following nusinersen treatment. (**H**) Individual immunoblot data for each iPSC line, including SMN and KIF5A (corresponding to Figure 3**I**) as well as KIF5B and KIF5C, across WT, SMA + non-targeting ASO, and SMA + nusinersen conditions. These data correspond to the group-averaged quantifications shown in Figure 3**I** and Supplemental Figure 4**G**. Quantified data in (**A**,**C**,**E**, and **G**) were derived from three biologically independent experiments performed in triplicate and analyzed using one-way ANOVA followed by Tukey's multiple comparisons test.

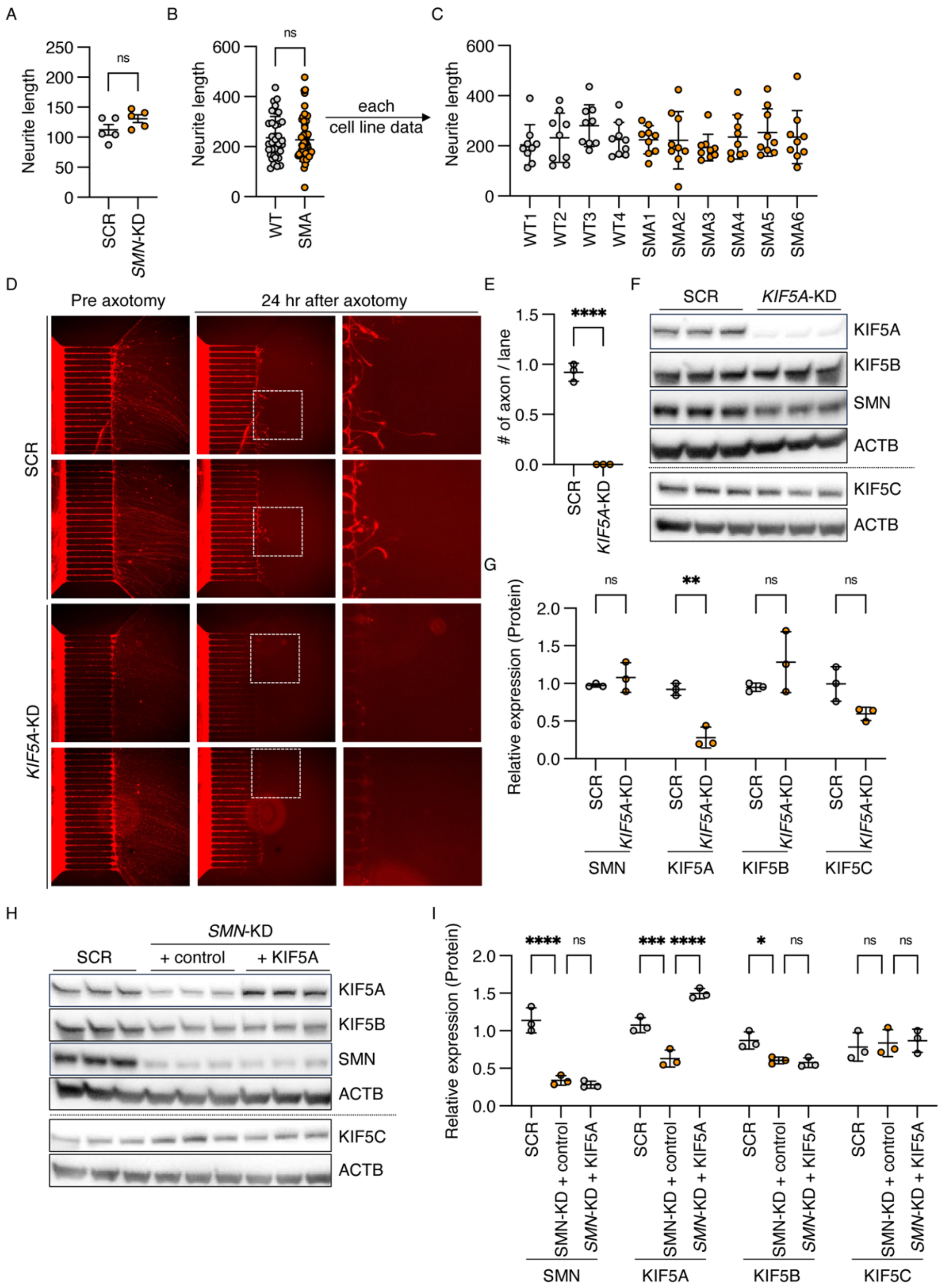

Supplemental Figure 5. KIF5A regulates axonal regeneration in i³Neurons

(**A**–**C**) Neurite length is not significantly altered by *SMN* knockdown (*SMN*-KD) in i³Neurons (i³Ns) or under SMA conditions. Neurite length (μm/cell) shows no significant differences in *SMN*-

KD i[3]Ns or SMA patient derived motor neurons (iMNs) compared with controls (scrambled shRNA; SCR for i[3]Ns (**A**) or WT for iMNs (**B**)). Cell line–specific data corresponding to (**B**) are shown in (**C**). (**D–G**) *KIF5A*-KD impairs axonal regeneration independently of other kinesin-1 family members. (**D**) Representative live-cell images of i[3]Ns cultured in microfluidic devices before and after axonal transection. Axons extend from the somatic compartment (left) into the axonal compartment (right). The length of each microchannel is 450 μm. (**E**) Quantification of regenerating axons per microfluidic channel following *KIF5A*-KD. Data represent the average number of regenerating axons per channel, normalized to control conditions (n = 3 independent experiments, with 2 images per experiment; 17–20 channels per image; >100 channels total). Statistical analysis was performed using an unpaired two-tailed t-test with Welch's correction. (**F** and **G**) Immunoblot analysis confirming efficient *KIF5A*-KD in i[3]Ns following 10 days of shRNA induction and demonstrating that *KIF5A*-KD does not alter the protein levels of other kinesin-1 family members. Representative immunoblots are shown in (**F**). Dashed lines indicate that lanes were derived from different membranes. Quantification is shown in (**G**). Data represent three independent experiments. Statistical analysis was performed using an unpaired two-tailed t-test with Welch's correction. (**H** and **I**) Immunoblot analysis confirming KIF5A overexpression in *SMN*-KD i[3]Ns, corresponding to experiments shown in Figure 4. Representative immunoblots are shown in (**H**). Dashed lines indicate that lanes were derived from different membranes. Quantification is shown in (**I**). Statistical analysis was performed using one-way ANOVA followed by Tukey's multiple comparisons test.

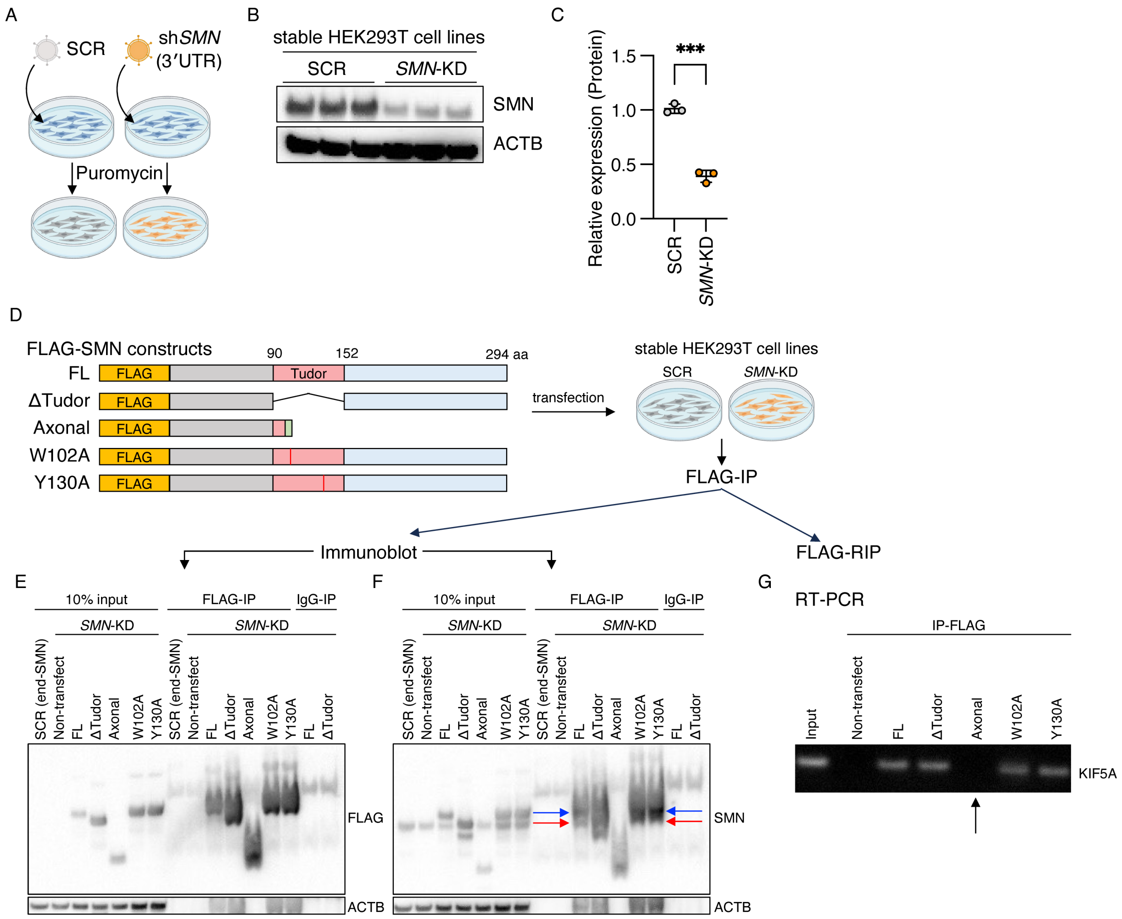

Supplemental Figure 6. Characterization of SMN–*KIF5A* mRNA association using SMN domain mutants

(**A**–**C**) Generation and validation of stable *SMN*-knockdown (KD) HEK293T cells used for luciferase reporter (Figure 5, **C** and **D**) and RNA immunoprecipitation (RIP) assays (panel **D**–**F**). (**A**) Experimental schematic. (**B**) Immunoblot results confirming efficient *SMN*-KD following lentiviral transduction and puromycin selection. (**C**) Quantification of SMN protein levels relative to control cells. Statistical analysis was performed using an unpaired two-tailed t-test with Welch's correction. (**D**–**G**) RIP assays using FLAG-tagged SMN constructs carrying Tudor domain deletions or point mutations (W102A and Y130A), as well as axon-SMN. (**D**) Experimental schematic of RIP assays using FLAG-tagged SMN constructs. (**E**) Immunoblot analysis of FLAG

immunoprecipitates detected with an anti-FLAG antibody, confirming efficient expression and immunoprecipitation of FLAG-tagged SMN constructs. Endogenous SMN is not detected in FLAG immunoblots. (**F**) Immunoblot analysis of the same FLAG immunoprecipitates detected with an anti-SMN antibody. Both FLAG-tagged SMN (blue arrow) and endogenous SMN (red arrow) are detected, indicating co-immunoprecipitation of endogenous SMN with FLAG-tagged constructs, except for axon-SMN. (**G**) RIP followed by RT-PCR analysis assessing association of SMN constructs with KIF5A mRNA. Truncated axonal SMN constructs failed to associate with KIF5A mRNA (black arrow in **G**), whereas Tudor domain–deficient or mutant SMN constructs retained detectable association. This residual association is likely mediated by co-immunoprecipitated endogenous wild-type SMN, which remains capable of binding KIF5A mRNA.

List of Supplemental Tables

Supplemental Table 1: Stem cell information

Supplemental Table 2: DEGs lists

Supplemental Table 3: Medium list

Supplemental Table 4: Antibodies list

Supplemental Table 5: Primers
