# Supplementary material for: KIF5A downregulation in spinal muscular atrophy links axonal regeneration defects with ALS
Source: JCI Insight. 2026 Mar 26;11(10):e197941. doi: 10.1172/jci.insight.197941 (PMC13232735; doi:10.1172/jci.insight.197941)

Figure 1F

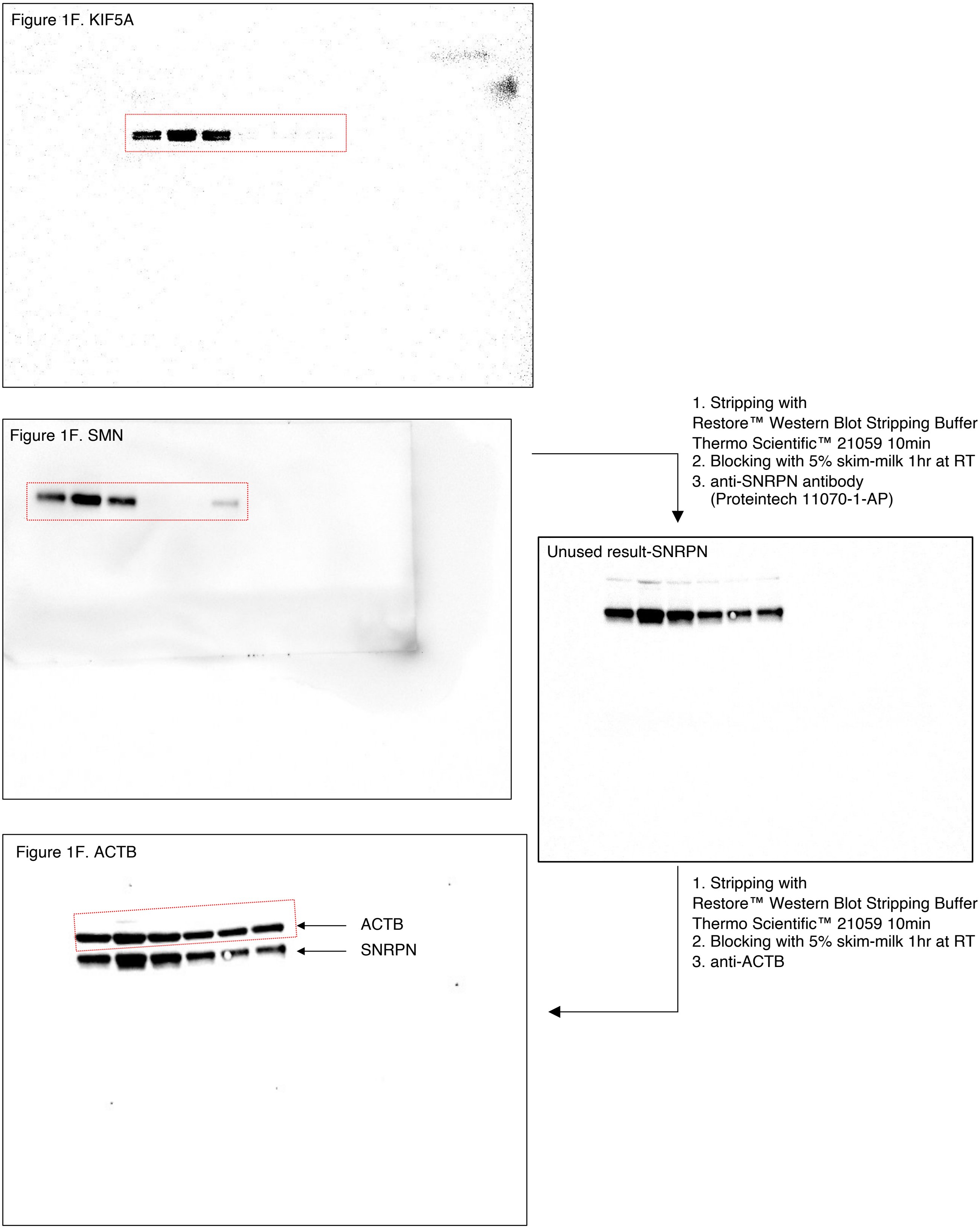
Figure 1F. KIF5A
Figure 1F. SMN
1. Stripping with
Restore™ Western Blot Stripping Buffer
Thermo Scientific™ 21059 10min
2. Blocking with 5% skim-milk 1hr at RT
3. anti-SNRPN antibody
(Proteintech 11070-1-AP)
Unused result-SNRPN
Figure 1F. ACTB
ACTB
SNRPN
1. Stripping with
Restore™ Western Blot Stripping Buffer
Thermo Scientific™ 21059 10min
2. Blocking with 5% skim-milk 1hr at RT
3. anti-ACTB

Figure 1H

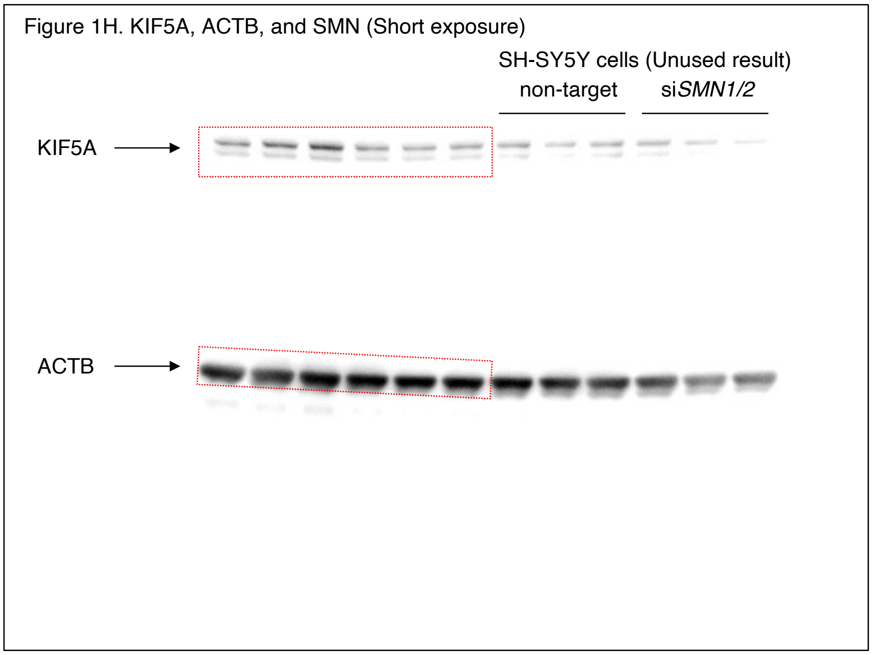

Figure 1H. KIF5A, ACTB, and SMN (Short exposure)
SH-SY5Y cells (Unused result)
non-target
siSMN1/2
KIF5A
ACTB

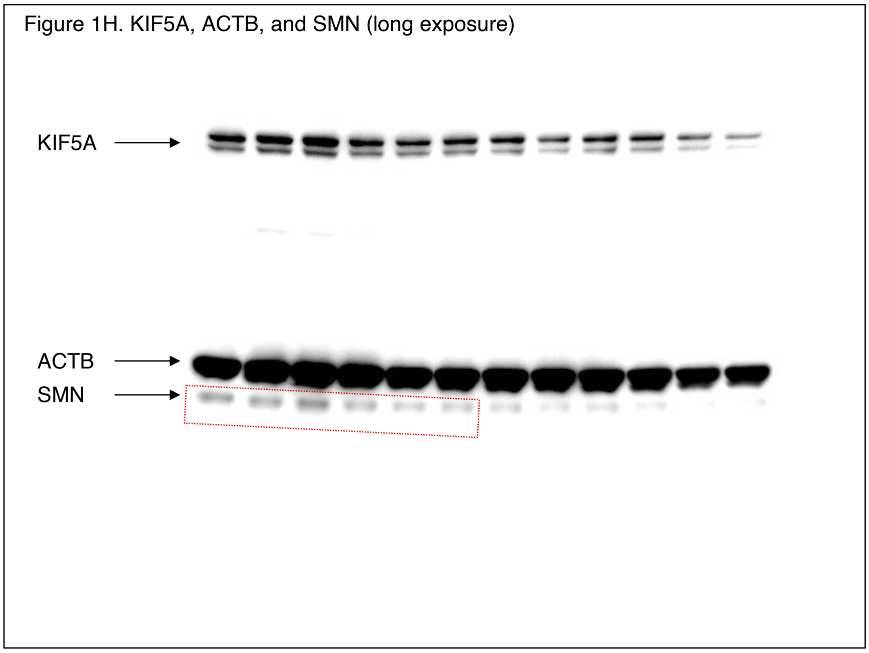

Figure 1H. KIF5A, ACTB, and SMN (long exposure)
KIF5A
ACTB
SMN

Figure 3E and Supplemental Figure 4B

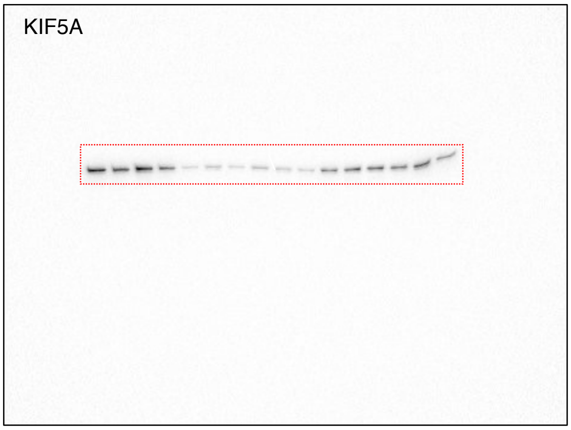

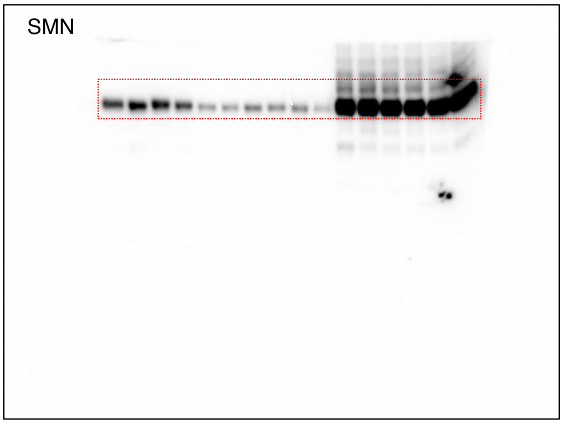

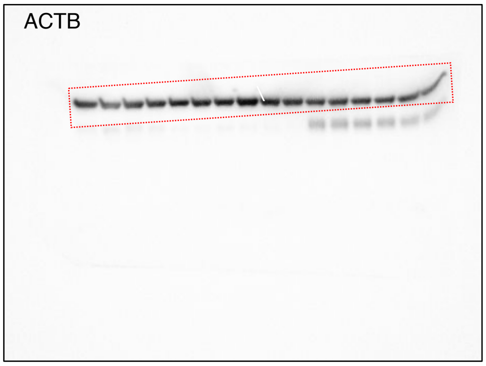

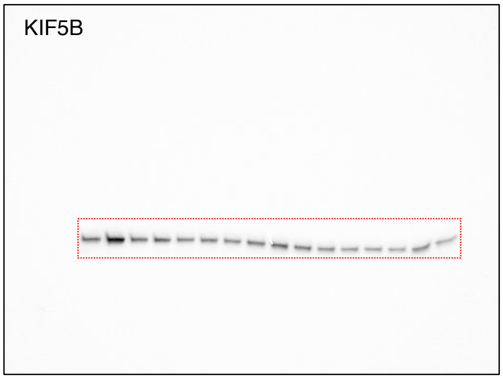

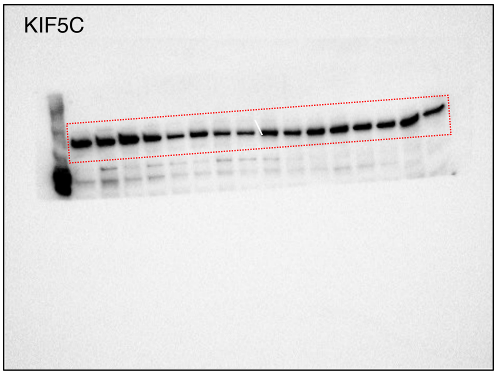

Figure 3H and Supplemental Figure 4F

Figure 3H. KIF5A

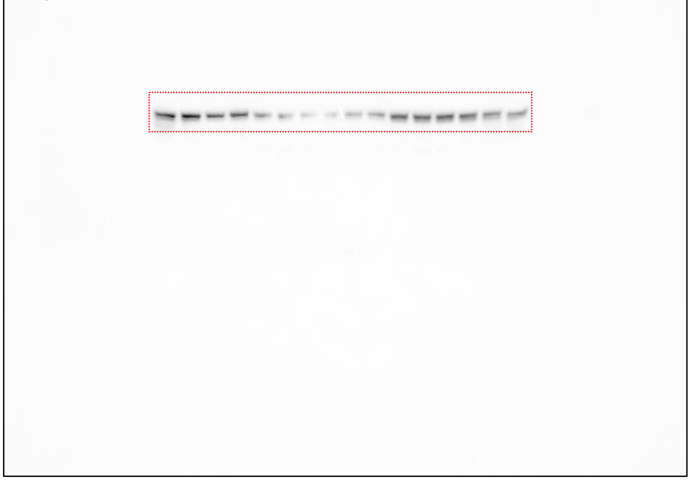

Figure 3H. SMN

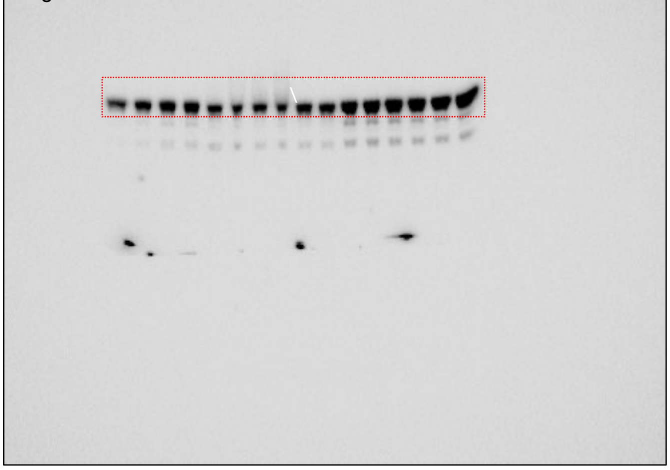

Figure 3H. ACTB

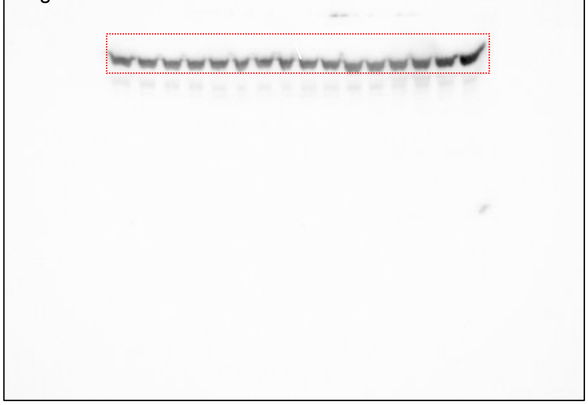

KIF5B

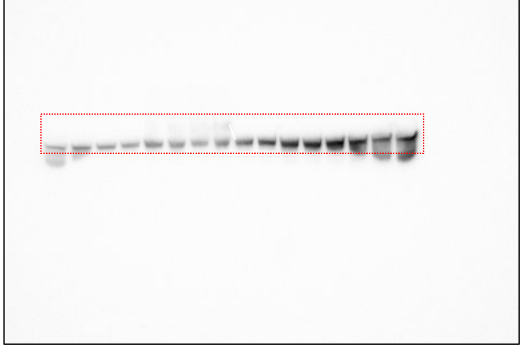

KIF5C

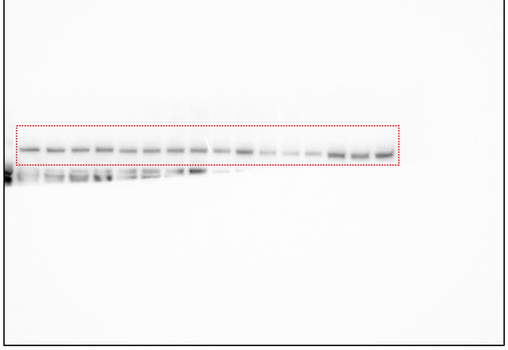

Supplemental Figure 4F SMN and ACTB

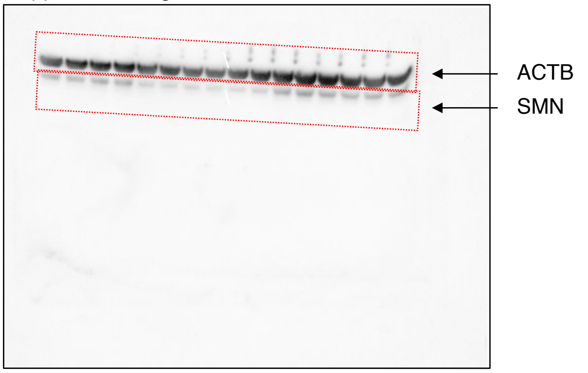

Figure 5

Figure 5E

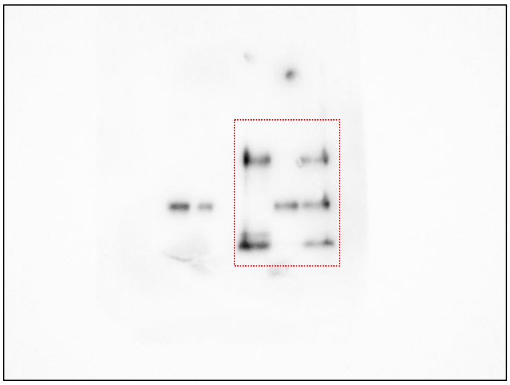

Figure 5H

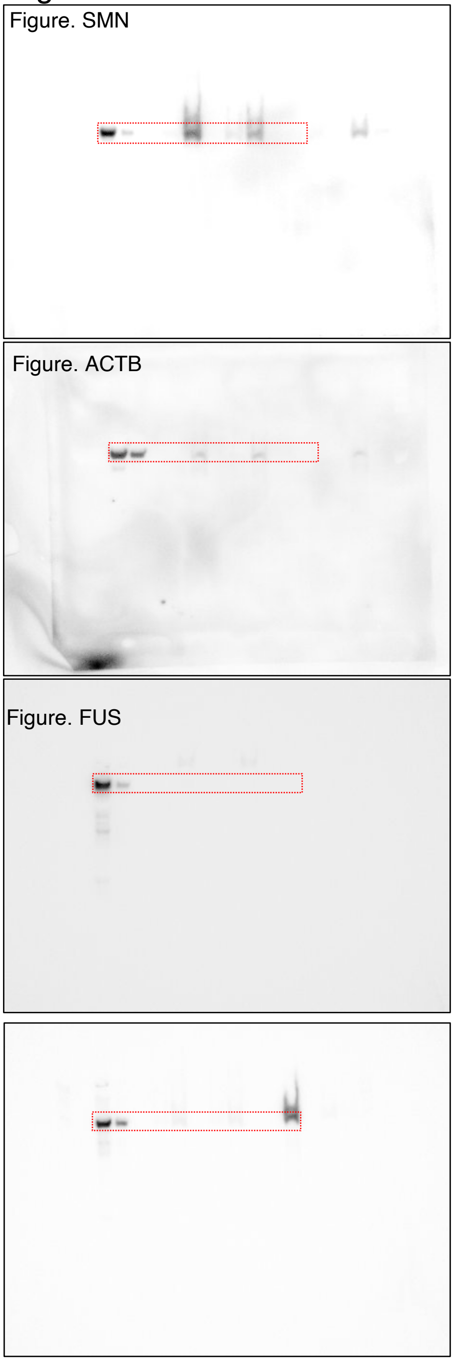

1. Stripping with
Restore™ Western Blot Stripping Buffer
Thermo Scientific™ 21059 10min
2. Blocking everyblot 1hr at RT
3. anti-ACTB antibody

1. Stripping with
Restore™ Western Blot Stripping Buffer
Thermo Scientific™ 21059 10min
2. Blocking everyblot 1hr at RT
3. anti-FUS antibody

1. Stripping with
Restore™ Western Blot Stripping Buffer
Thermo Scientific™ 21059 10min
2. Blocking everyblot 1hr at RT
3. anti-TDP-43 antibody

Supplemental Figure 1B

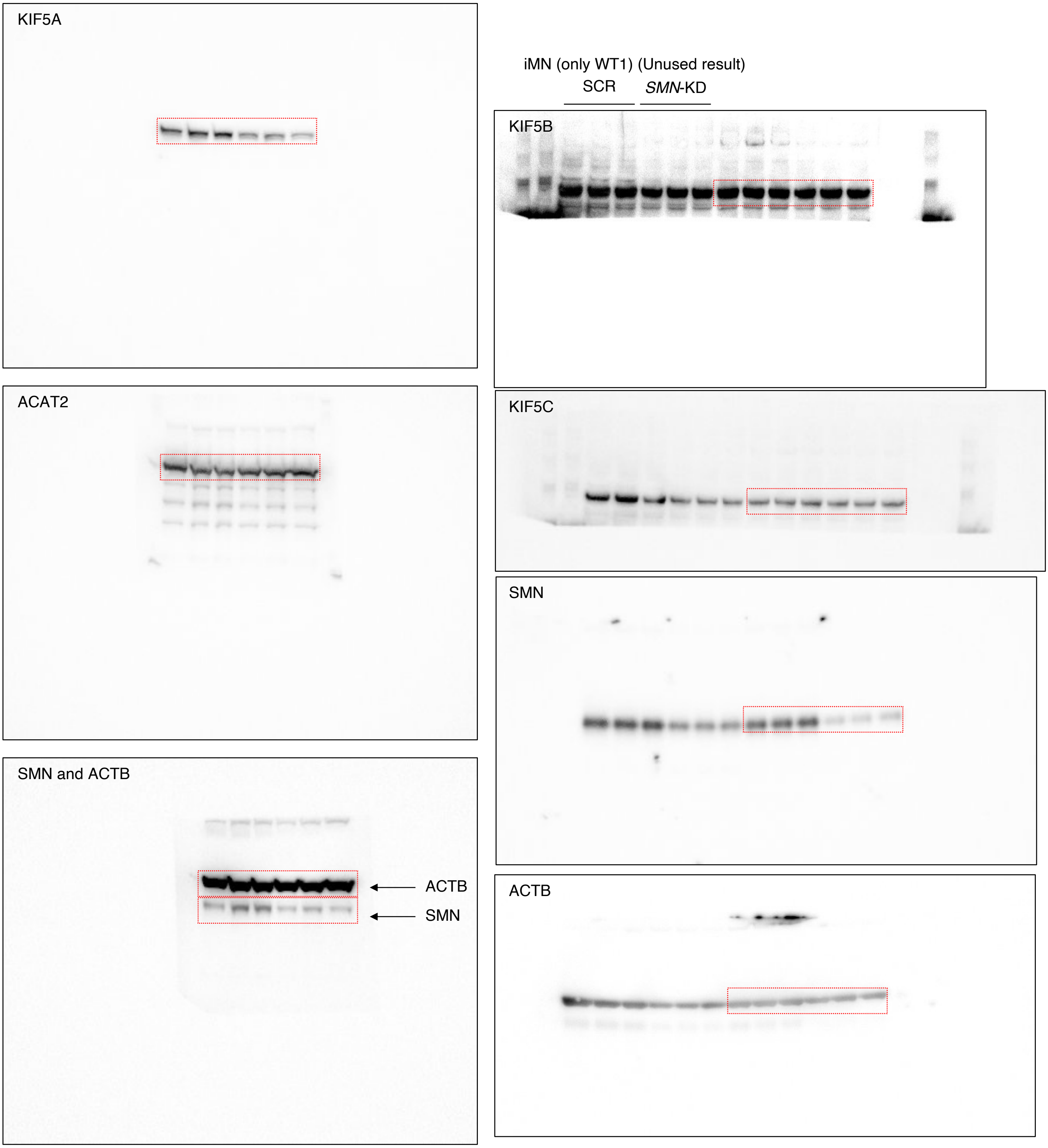

Supplemental Figure 1E

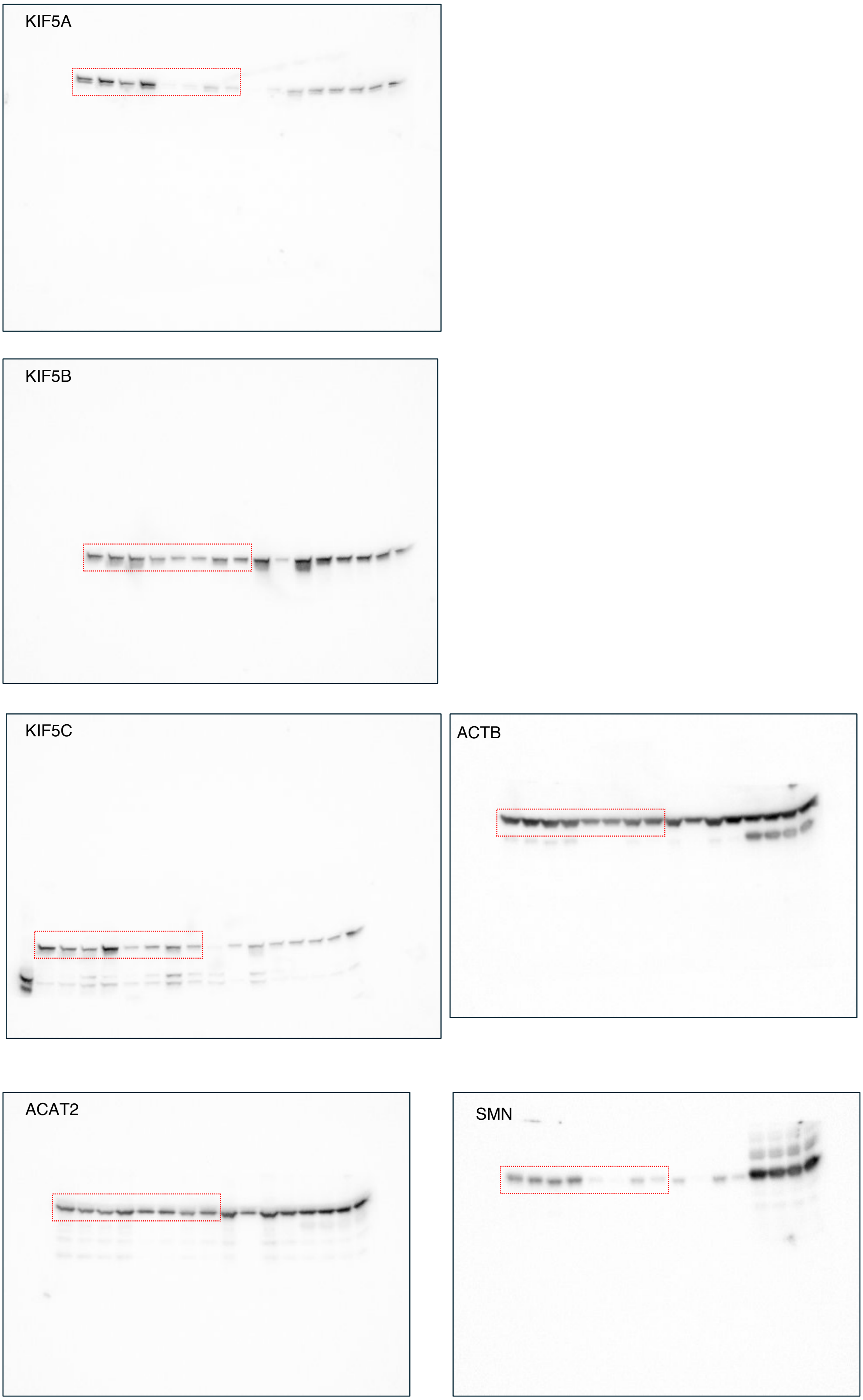

Supplemental Figure 5

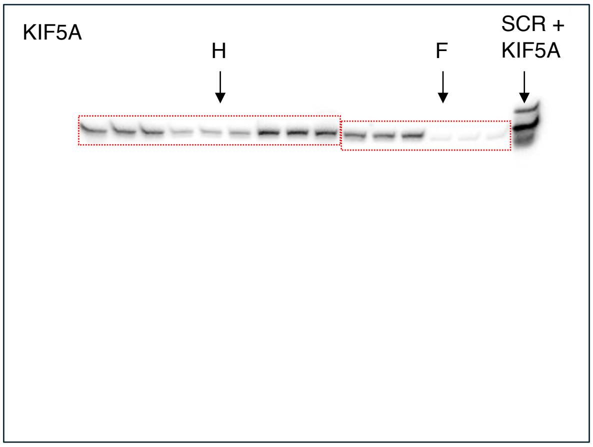

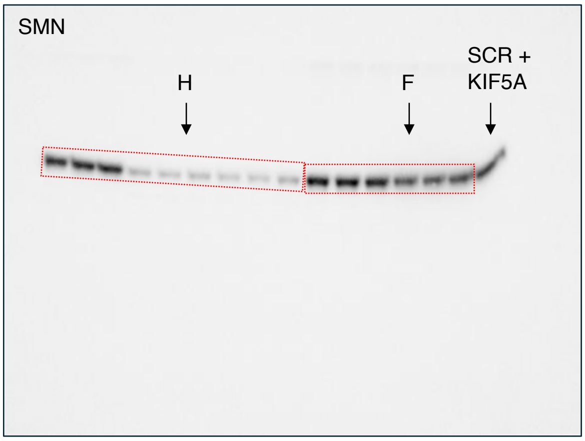

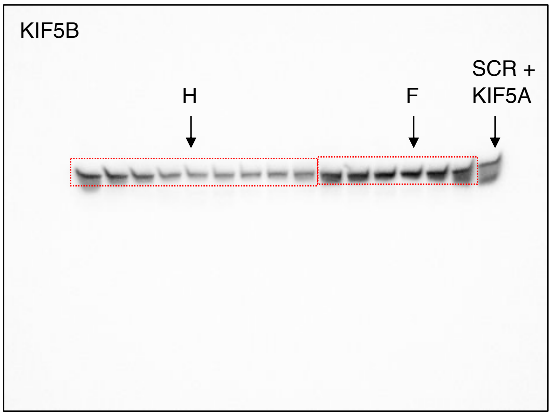

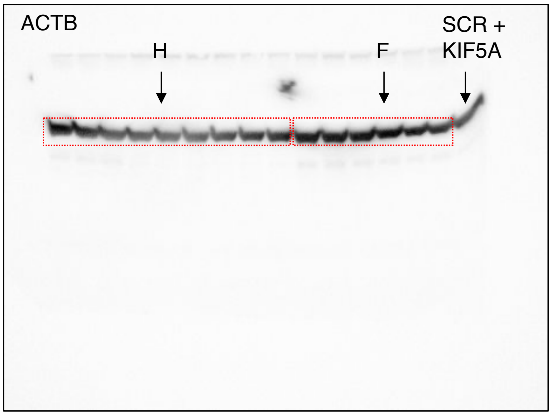

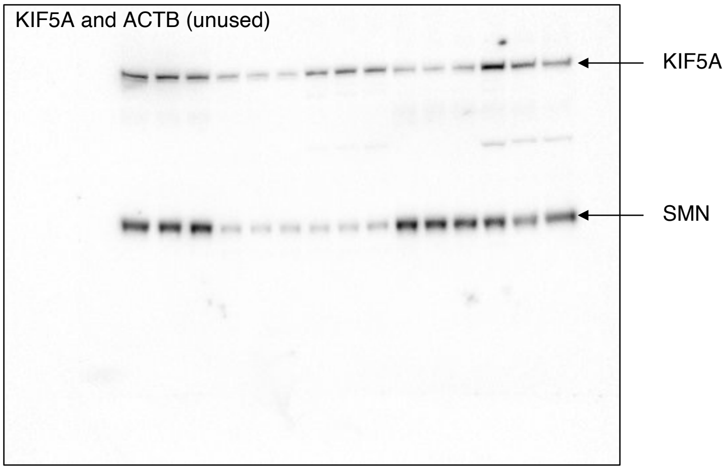

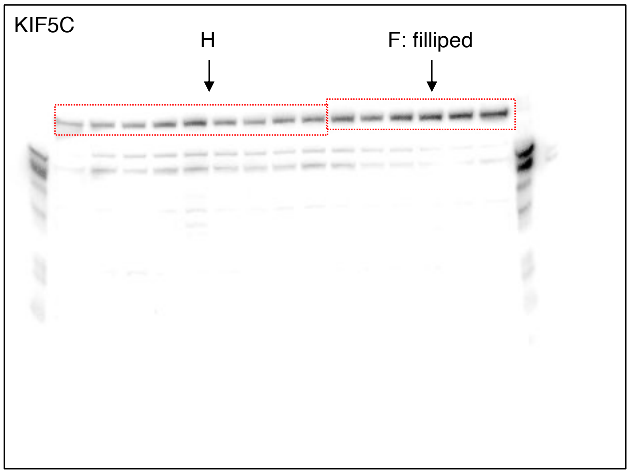

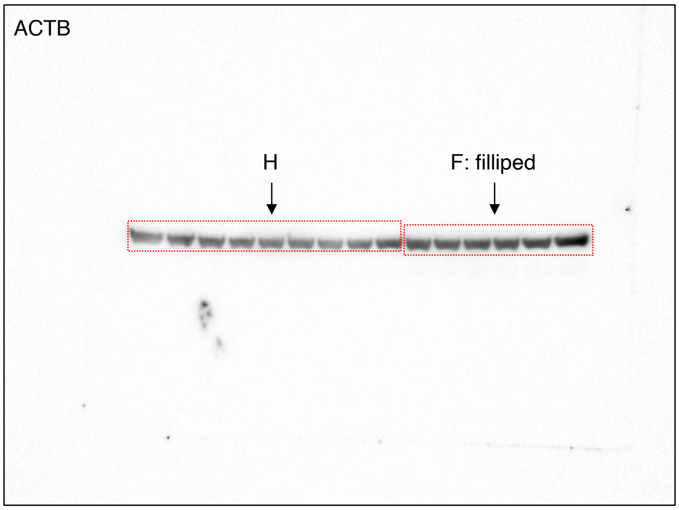

Supplemental Figure 6B

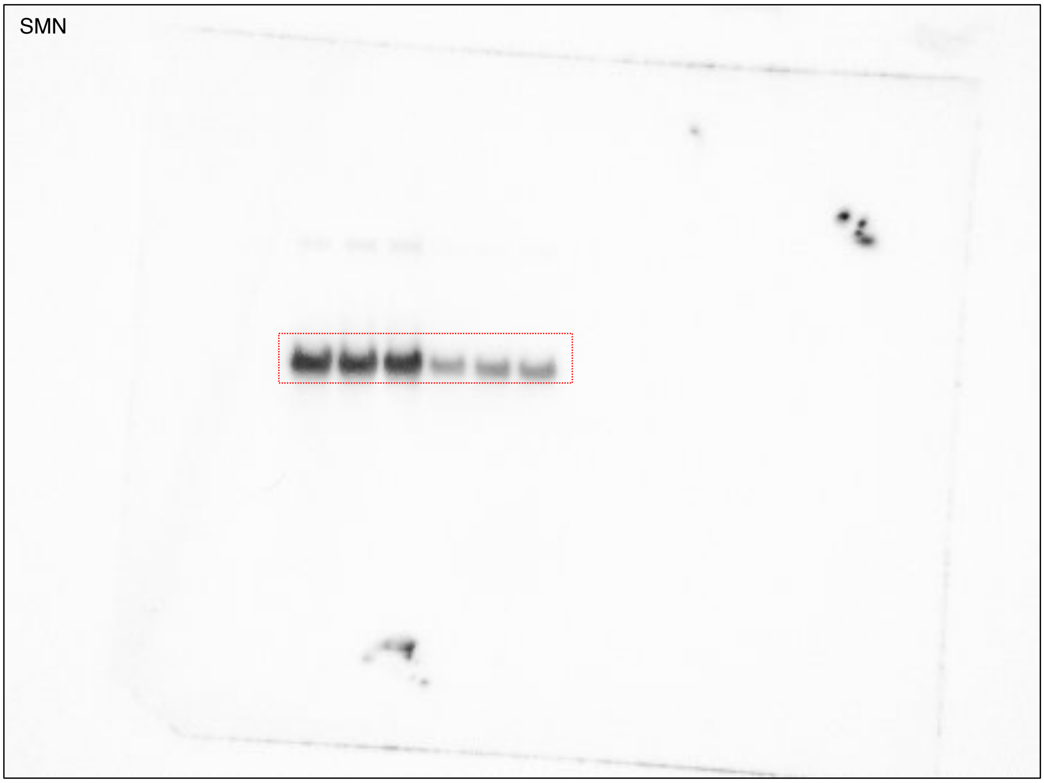

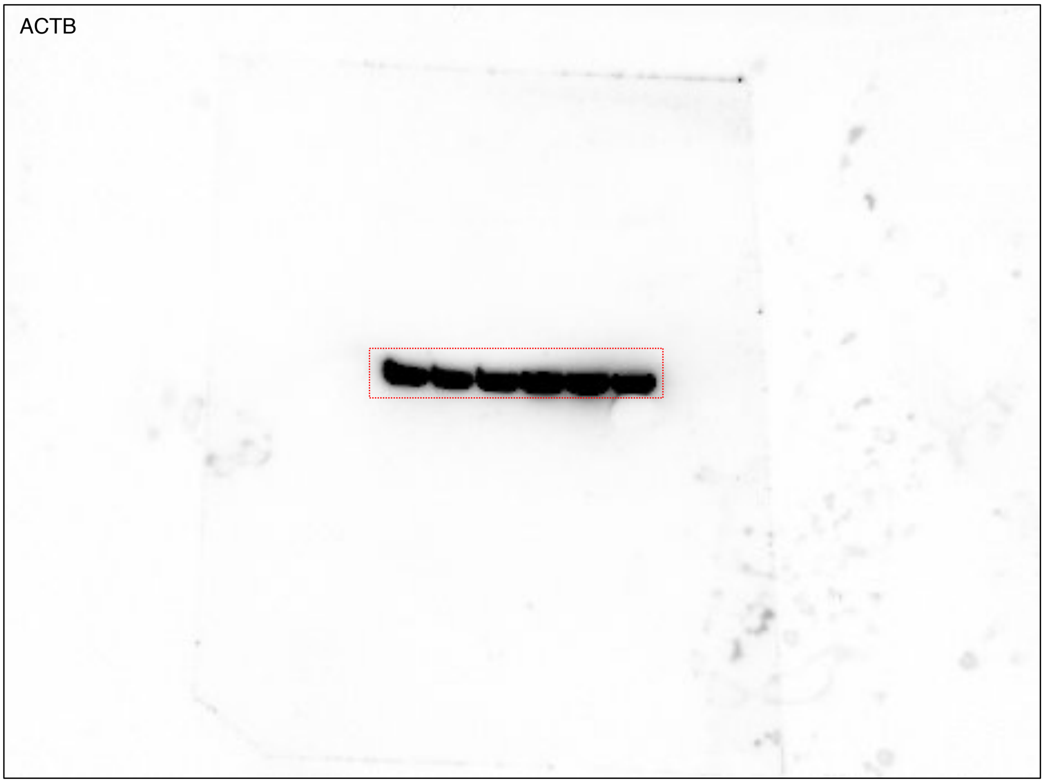

Supplemental Figure 6

Supplemental Figure 6E

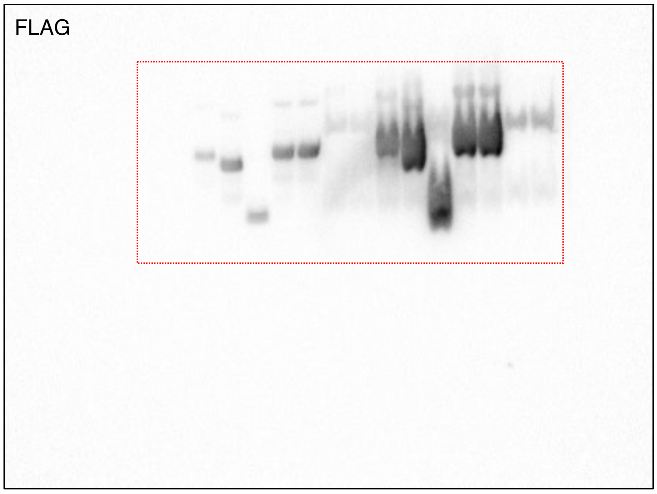

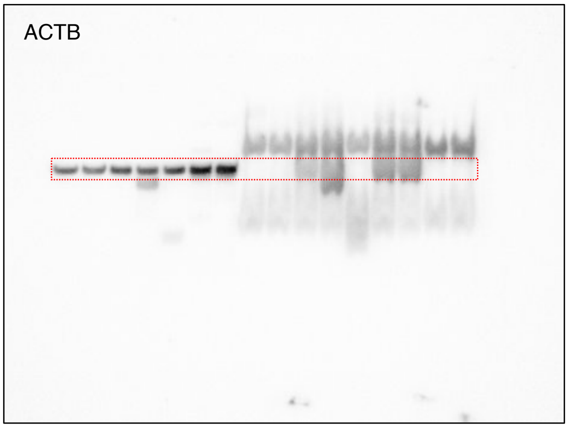

Supplemental Figure 6F

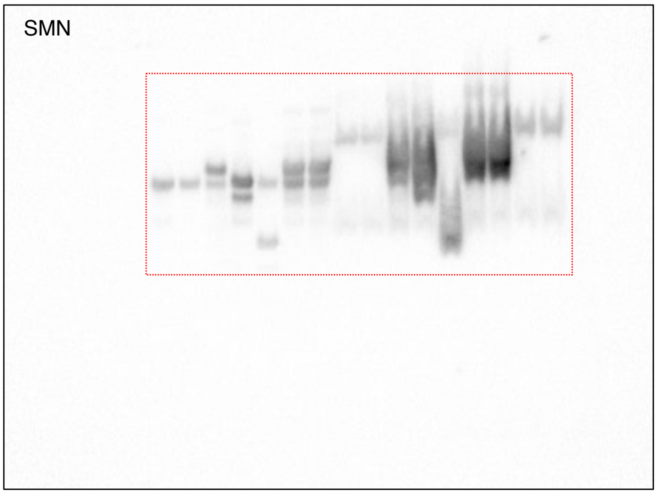

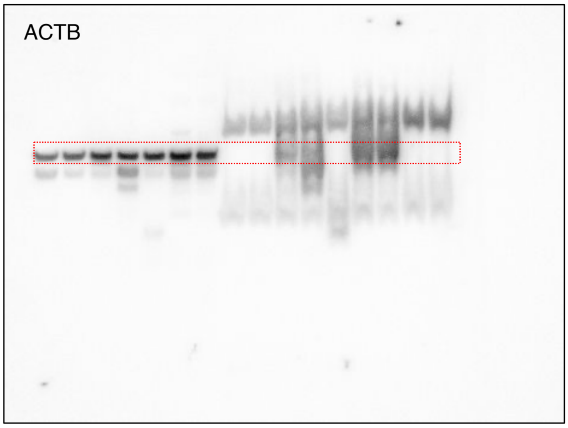

Supplement: Unedited blot and gel images [file jciinsight-11-197941-s234.pdf]
